# Hybrid EEG–EMG intention decoding for real-time triggering of a lower-limb exoskeleton

**DOI:** 10.3389/fnbot.2026.1914651

**Published:** 2026-09-03

**Authors:** Ke Wang, Wenshan Li, Danyang Ding, Lei Wang, Yue Sun, Hongbo Zhao, Weijun Gong

**Affiliations:** 1 The Third People’s Hospital of Jincheng, Jincheng, Shanxi, China; 2 Capital Medical University, Beijing, China; 3 Beijing Rehabilitation Hospital, Capital Medical University, Beijing, China; 4 RoboCT Technology Development Co., Ltd, Hangzhou, China

**Keywords:** BiLSTM, electroencephalography (EEG), electromyography (EMG), gait intent detection, hybrid BCI, lower-limb exoskeleton, real-time control, sensorimotor rhythms

## Abstract

**Background:**

Wearable lower-limb exoskeletons have the potential to support intention-driven control of lower-limb exoskeletons, but existing control strategies often rely on mechanical or manual triggers that fail to capture user intent.

**Method:**

Subjects were recruited from 23/9/2024 to 10/2/2025. A BiLSTM detector was pretrained on a dataset collected from 50 healthy volunteers (45 for training, 5 for independent testing) using bilateral surface EMG recordings from six lower-limb muscles (12 EMG channels), 16-channel EEG, and hip–knee kinematics. Seven naive participants then completed ten 20-m outward-and-return walking trials (10 m outward and 10 m return) under each of three control modes (EMG, EEG, hybrid). Primary outcomes were triggering latency and classification accuracy. Triggering latency was defined as the time interval between the onset of the gait-transition event and the activation of the exoskeleton assistance command. This latency included the observation delay introduced by the sliding window, feature extraction time, BiLSTM inference time, and communication delay between the decoder and the exoskeleton controller. Usability was assessed with donning/doffing times and QUEST 2.0.

**Results:**

The hybrid BiLSTM detector achieved higher classification accuracy (left: 91.3%; right: 86.6%) and shorter mean per-step triggering latency (left: 0.29 s; right: 0.28 s) than either EMG-only (mean 0.33 s) or EEG-only approaches (mean 0.30 s). Hybrid EEG–EMG fusion therefore improved decoding performance while reducing triggering latency compared with unimodal decoding strategies. The latency reduction relative to EMG-only control corresponded to a large effect size (Cohen’s d ≈ 0.88). Hybrid sessions also yielded shorter total session time (mean 32.1 min). Usability metrics demonstrated acceptable donning/doffing times and favorable QUEST 2.0 scores (mean 32.0/40).

**Conclusion:**

These proof-of-concept results demonstrate that BiLSTM-based fusion of EEG and EMG improves responsiveness and classification reliability for exoskeleton assistance. These findings underscore the contribution of EEG-derived sensorimotor features and EMG information for intention-related gait transition detection within a multimodal real-time exoskeleton control framework. We discuss limitations related to sample size, artifact validation, and generalizability and identify next steps for patient studies and ergonomic optimization.

## Introduction

Lower-limb exoskeletons have emerged as a promising technology for assisting locomotion and enhancing mobility in individuals with motor impairments (Louie et al., 2021). A critical challenge in exoskeleton control is accurately identifying the user’s movement intention and delivering assistance at the appropriate timing. Conventional exoskeleton systems commonly rely on kinematic thresholds, joint angles, foot switches, or force sensors to trigger assistance. Although these approaches are relatively simple and reliable, they are inherently reactive because assistance is initiated only after detectable biomechanical events have occurred. Such delays may reduce the synchronization between the user and the robotic device and limit the naturalness of movement. Real-time triggering latency is a critical determinant of user-exoskeleton synchronization because delayed assistance may reduce movement naturalness, compromise intention-assistance matching, and decrease user engagement during training.

To improve human-robot interaction, increasing attention has been directed toward intention-related movement decoding using neural signals (Soekadar et al., 2015; López-Larraz et al., 2016). Electroencephalography (EEG) provides a non-invasive means of monitoring cortical activity associated with motor planning and execution before overt movement occurs. Previous studies have demonstrated that movement-related cortical oscillations, particularly in the alpha (8–13 Hz) and beta (13–30 Hz) frequency bands, exhibit characteristic modulations during gait initiation and locomotion (Petersen et al., 2012; Wagner et al., 2016). These sensorimotor rhythm (SMR) modulations have been observed not only during discrete motor imagery paradigms but also during actual locomotor tasks, including gait initiation, walking, and lower-limb movement execution. Mu-band desynchronization has been associated with sensorimotor cortical activation related to movement preparation, whereas beta-band modulation has been linked to motor execution, postural regulation, and proprioceptive feedback processing. These neural signatures have therefore been investigated as potential biomarkers for intention-related exoskeleton control. Recent studies using online or pseudo-online EEG decoding approaches have further demonstrated the feasibility of detecting locomotor states and movement intentions from cortical signals, although motion artifacts and reduced signal-to-noise ratio remain major challenges during mobile walking conditions.

However, EEG-based decoding faces several challenges. Neural signals recorded during walking are susceptible to motion artifacts, muscle contamination, and environmental noise. Furthermore, cortical activity alone may not fully capture the peripheral execution of movement, potentially limiting decoding robustness in real-world locomotor tasks.

Electromyography (EMG), in contrast, directly reflects muscle activation and has been widely used for gait-phase detection and exoskeleton control. Compared with EEG, EMG generally exhibits higher signal-to-noise ratios and stronger associations with biomechanical events. Previous studies have extensively investigated EMG-based gait phase recognition, locomotor event detection, and intention prediction (Calabrò et al., 2021) for assistive devices. EMG signals from lower-limb muscles have been successfully used to identify gait transitions, estimate locomotor states, and trigger robotic assistance. Nevertheless, EMG primarily reflects peripheral motor execution and often becomes detectable only shortly before or during movement onset. Because EMG represents downstream neuromuscular activation rather than preceding motor intention, EMG-based control may provide highly reliable but relatively reactive information, which may limit its ability to anticipate upcoming gait transitions.

Given the complementary characteristics of EEG and EMG, multimodal decoding approaches have attracted increasing interest. EEG-derived sensorimotor features may provide information associated with movement preparation and intention-related transitions, whereas EMG captures peripheral neuromuscular activation related to movement execution. Integrating these modalities may improve the temporal precision and reliability of gait-transition detection by combining neural and muscular information compared with either modality alone. Recent studies have suggested that multimodal neural-muscular fusion can enhance movement decoding performance in robotic rehabilitation and assistive device applications (Garro et al., 2021; De Seta and Romeni, 2025). However, whether multimodal EEG–EMG integration can provide reliable and timely information associated with gait-intention-related transitions during real-time exoskeleton-assisted walking remains insufficiently explored.

Beyond decoding performance, understanding the neurophysiological interactions underlying human-robot locomotion is equally important. During walking, cortical activity and muscle activation are dynamically coordinated through the corticospinal pathway (Artoni et al., 2017). Cortico-muscular coupling (CMC) provides a quantitative measure of functional connectivity between the motor cortex and peripheral muscles and has been widely used to investigate locomotor control (Mima and Hallett, 1999). Previous studies have reported phase-dependent modulation of cortical oscillations and CMC during natural walking, suggesting that neural control strategies vary across different gait phases (Petersen et al., 2012). Whether similar neural coordination patterns are preserved during exoskeleton-assisted walking remains incompletely understood.

Characterizing these neural dynamics may not only improve our understanding of human-robot interaction but also facilitate the development of physiologically informed control strategies. Specifically, identifying gait-phase-dependent cortical and muscular features may provide useful information for intention-aware exoskeleton triggering and adaptive assistance.

Therefore, the primary objective of this study was to investigate whether multimodal EEG–EMG fusion can improve the temporal precision and reliability of gait-intention detection for real-time exoskeleton triggering. To achieve this goal, we first characterized phase-specific cortical oscillations, muscle activation patterns, and cortico-muscular coupling during hybrid exoskeleton-assisted walking in healthy participants. We then developed a multimodal gait-event decoding framework based on EEG and EMG signals using a bidirectional long short-term memory (BiLSTM) network and evaluated its performance for predicting intention-related gait transitions. We hypothesized that (1) exoskeleton-assisted walking would exhibit distinct phase-dependent cortical and cortico-muscular modulation patterns, and (2) multimodal EEG–EMG fusion would improve decoding accuracy, reduce triggering latency, and enhance real-time exoskeleton triggering performance compared with single-modality approaches.

Although previous studies have investigated EEG–EMG fusion for upper-limb movement decoding, motor rehabilitation, and assistive device control, the application of multimodal neural-muscular fusion for real-time gait intention detection during powered lower-limb exoskeleton walking remains insufficiently explored. Furthermore, many previous decoding studies have focused primarily on offline classification performance, whereas practical exoskeleton control requires not only accurate decoding but also timely translation of user intention into robotic assistance. Therefore, the present study specifically addresses the challenge of converting multimodal neural-muscular information into real-time gait-event triggering during human-robot interaction.

The findings may contribute to the development of intention-driven and neurophysiological informed exoskeleton control systems by providing evidence that EEG-derived sensorimotor features can contribute to intention-related gait transition detection when integrated with EMG-based neuromuscular information.

## Method

### Exoskeleton system and specifications

**Figure 1:**
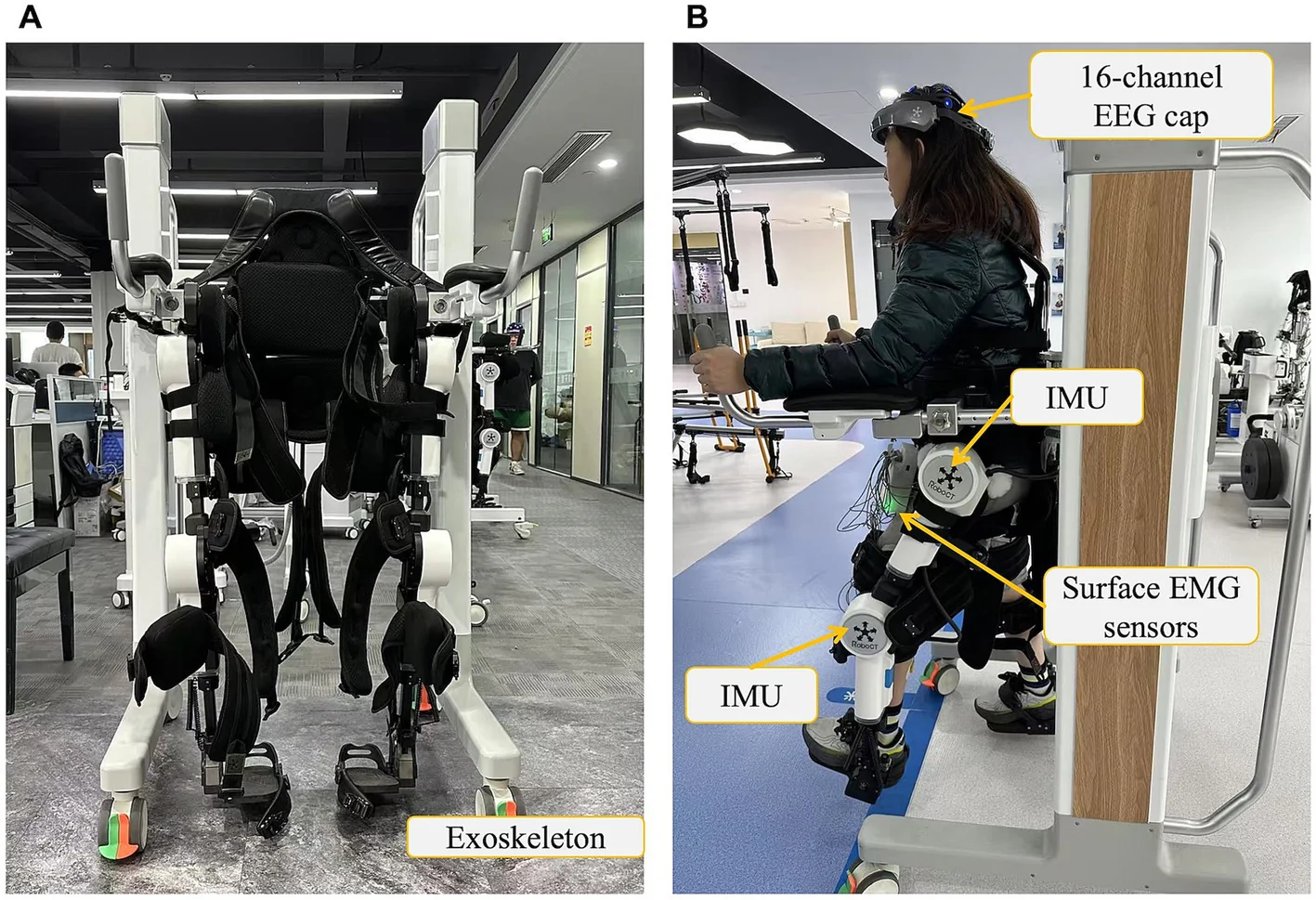
Experimental setup **(A)** wearable exoskeleton; and **(B)** participant wearing a 16-channel EEG cap, bilateral surface EMG sensors.

**Figure 2:**
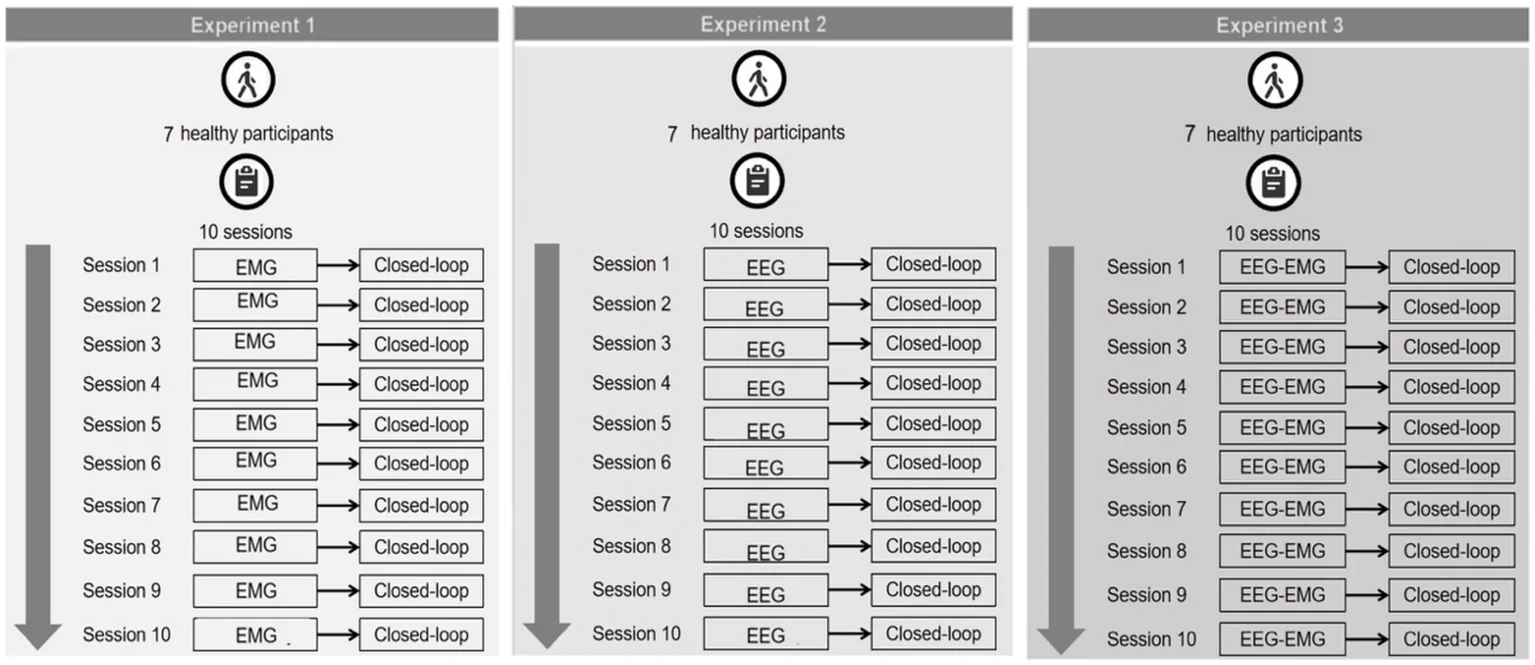
Schema of the three experiments that were conducted in the present study.

**Figure 3:**
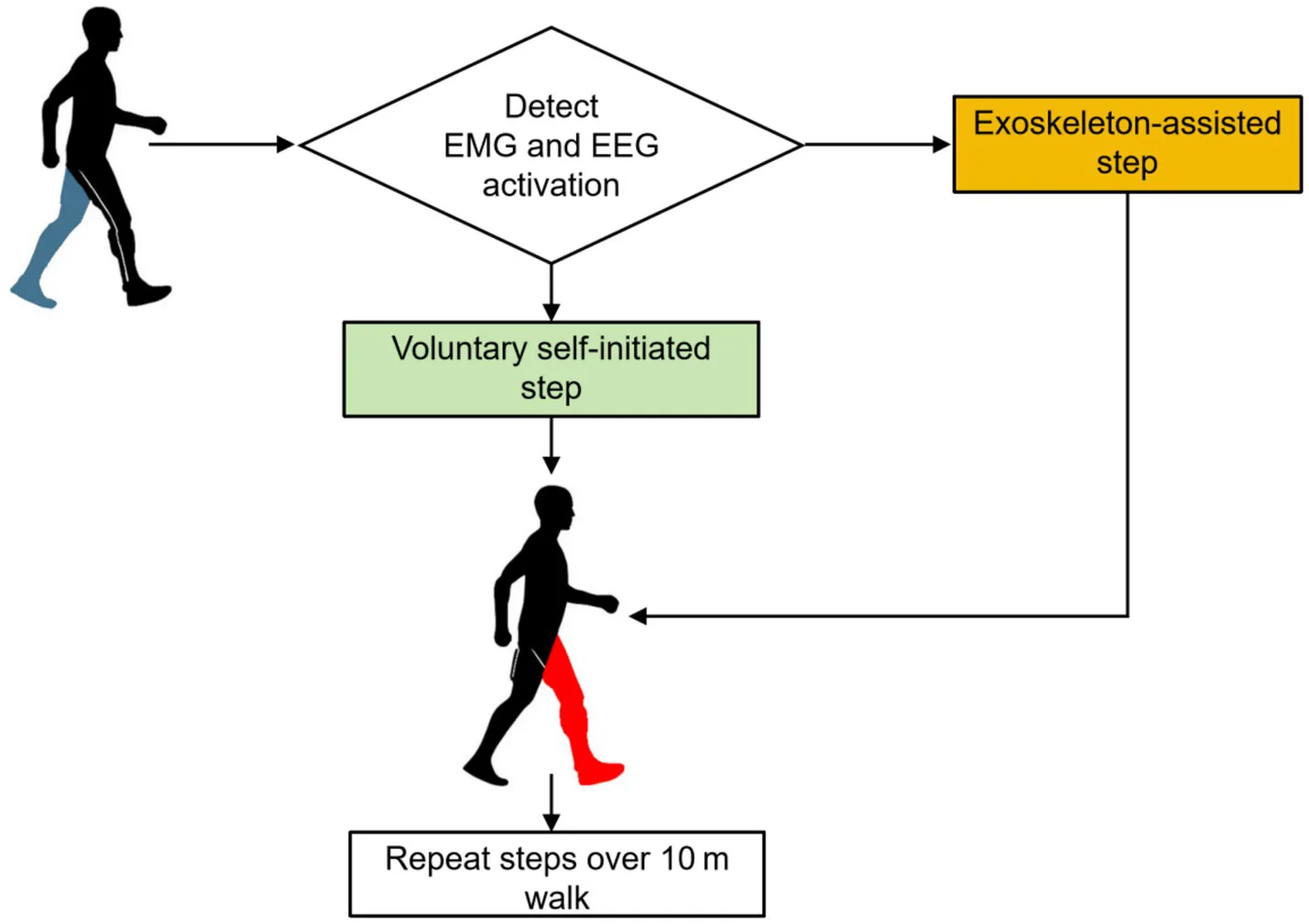
EMG–EEG driven gait assistance flow.

A powered hip–knee lower-limb exoskeleton was used in this study. The system consisted of bilateral hip and knee actuators mounted on an external walking aid frame to ensure safety during overground and walking-in-place experiments. The total system mass was approximately 100 kg, which included the actuation units, structural components, onboard controllers, batteries, and the walking aid frame. The exoskeleton itself was not fully self-supporting and relied on the frame to offload weight, making it suitable for laboratory-based feasibility and proof-of-concept evaluation rather than free-living use (Figures 1–3).

Each hip and knee joint was actuated in the sagittal plane. Ankle joints were not actively actuated and were mechanically constrained to a neutral position through the footplate; no active dorsiflexion or plantarflexion assistance was provided. Joint encoders were embedded at the hip and knee to record joint angles in real time. Maximum assistive torque was predefined and limited by software safety constraints to prevent excessive joint loading. Emergency stop buttons and torque saturation limits were implemented as safety interlocks throughout all experiments.

EEG data were acquired wirelessly via a 16-channel portable system with electrodes placed according to the international 10–20 montage. The recorded EEG channels included Fp1, Fpz, Fp2, F3, F4, F7, F8, Fz, C3, Cz, C4, P3, P4, Pz, O1, and O2, covering bilateral frontal, central, parietal, temporal, and occipital regions with particular emphasis on the sensorimotor areas (C3, Cz, and C4). Simultaneously, surface EMG signals were recorded using a wireless acquisition unit, with electrodes bilaterally over the gluteus maximus, gluteus medius, quadriceps femoris, biceps femoris, gastrocnemius, and tibialis anterior. Therefore, bilateral recordings resulted in 12 EMG channels in total. All bio signals were captured in synchrony with the exoskeleton’s real-time kinematic (hip and knee angles) and kinetic (actuator torque) recordings, enabling multimodal data fusion in the hybrid control algorithm.

### Participants

Two experimental datasets were used in this study. First, an offline dataset was collected from 50 healthy adults to train and validate the intention detection model. These data were used exclusively for model development and did not involve exoskeleton-assisted walking. Second, seven able-bodied volunteers (2 males and 5 females; mean age 32.0 ± 4.8 years) participated in the online closed-loop exoskeleton experiments. All participants had no history of neurological or musculoskeletal disorders. The study protocol was approved by the local ethics committee, and all participants provided written informed consent prior to participation. Participants were not blinded to the control mode (EMG, EEG, or hybrid), as differences in system behavior and donning procedures made blinding impractical. The order of control modes was fixed across participants, and trial order was later included as a covariate in the statistical analysis to partially account for learning and fatigue effects.

The study enrolled 50 healthy participants (35 males; mean age: 32.9 ± 5.5 years; mean height: 168.8 ± 8.2 cm; mean body mass: 67.7 ± 11.5 kg), who had no history of neurological, musculoskeletal, cardiovascular, visual, or dermatological disorders that might limit the use of the exoskeleton or influence experimental results. Participants were recruited between 23 September 2024 and 10 February 2025. All participants were right-handed according to the Edinburgh Handedness Questionnaire (EHQ) and had no prior experience with robotic gait assistance. Data collection was performed in accordance with the Declaration of Helsinki. All participants were fully informed about the objectives, procedures, and potential risks of the study and provided written informed consent prior to participation. Ethical approval was obtained from the Institutional Review Board of Beijing Rehabilitation Hospital (Approval No. 2023bkky-083). Further demographic information is provided in Table 1.

**Table 1:** Demographic characteristics of participants (*n* = 7).

Participant	Gender	Age (years)	Height (cm)	Weight (kg)
1	F	25	158	49
2	F	27	163	61
3	M	34	170	70
4	M	30	172	53
5	F	38	158	61
6	F	35	166	60
7	F	43	159	48
Mean ± SD	—	32.0 ± 4.8	163.7 ± 5.8	57.4 ± 7.8

### Experimental design

The study comprised three stages: (1) offline model training and validation, (2) online feasibility testing, and (3) closed-loop comparison of control modes. EEG and EMG data from 50 participants were used for offline model development, while an additional group of seven participants was recruited for online validation and real-time control evaluation.

For offline training, EEG and EMG data collected from 50 healthy participants during walking tasks were segmented and labeled to train the BiLSTM-based intention detector. During offline data collection, participants performed overground walking trials at a self-selected comfortable speed. Each participant completed repeated walking sequences consisting of continuous walking and rest intervals. Participants were instructed to walk naturally while maintaining a comfortable pace. Each offline recording session consisted of multiple walking trials, with each trial including continuous overground walking followed by a resting interval. The total walking recording duration was approximately 10 min per participant, and each participant completed approximately 200 gait cycles after preprocessing and artifact rejection. No treadmill assistance or external support was used during offline data acquisition. Participants wore the lower-limb exoskeleton throughout the recording session, while EEG, EMG, and exoskeleton kinematic signals were synchronously recorded. The recorded EEG, EMG, and kinematic signals were segmented into gait cycles according to hip joint trajectories for subsequent labeling.

For online experiments, each participant completed walking tasks under three control conditions: EMG-only, EEG-only, and hybrid EEG–EMG control. Before data collection, all participants underwent a familiarization session to become accustomed to the exoskeleton, the walking environment, and the three control modes. Participants were instructed to walk at a self-selected comfortable speed. Each trial consisted of a 20-m outward-and-return walking route, including a 10-m outward path and a 10-m return path, with each condition including ten trials, resulting in a total of 30 trials per participant. Each trial lasted approximately 1 min, including walking, turning, and stabilization periods. Rest periods of approximately 1 min were provided between trials and conditions to minimize fatigue. The online pilot experiments focused on stance-to-swing transition detection during steady-state walking. Gait initiation and termination were not considered within the scope of this study.

EMG: Bilateral surface EMG from six lower-limb muscles was band-pass filtered and envelope-extracted before input to a pretrained BiLSTM detector. Each step proceeded identically: (1) monitor hip angle until it entered the −10° to 40° window and reached peak extension; (2) detect EMG activation when the envelope exceeded three times the moving baseline standard deviation; (3a) if activation occurred, deliver powered assistance for the stance-to-swing transition; (3b) otherwise, the participant self-initiated the step; and (4) repeat until 20-m outward-and-return walking trial (10 m outward and 10 m return) completion. Trial duration was recorded as an additional metric.

EEG: Participants donned a 16-channel EEG cap (C3 reference) without EMG probes. A pretrained asynchronous EEG decoder continuously computed band-power in *θ* (4–7 Hz), *μ* (8–13 Hz), low *β* (14–20 Hz), and high *β* (21–30 Hz). Prior to each walking trial, a resting baseline period was recorded and used as the reference for ERD-related feature calculation. The same baseline normalization parameters obtained during calibration were applied during online decoding without further adaptation. During online walking, EEG band-power features were computed relative to this predefined baseline period and continuously provided to the decoder for stance-to-swing intention detection. The EEG features and model parameters were determined based on the calibration data collected during the offline training phase. The decoder generated a classification probability representing the likelihood of an intention-related gait transition. When the predicted probability exceeded the predefined decision threshold, the exoskeleton assistance command was triggered; otherwise, the participant completed the transition through self-initiation without robotic assistance. Trial time and rest intervals matched the EMG-only protocol.

Hybrid EEG–EMG: In the hybrid condition, EEG band-power features and EMG envelope features were continuously acquired and jointly processed by a pretrained multimodal BiLSTM decoder. EEG and EMG signals were not evaluated using independent detection rules; instead, both modalities were concatenated into a unified feature representation and provided as input to a single BiLSTM model. The decoder generated a classification probability representing the likelihood of a stance-to-swing gait transition. When the output probability exceeded the predefined decision threshold within the control window, powered assistance was triggered. If no valid detection occurred, participants completed the step through self-initiation without robotic assistance. Trials, rest periods, and completion-time measurements followed the same protocol as the unimodal conditions.

### Online performance evaluation

Triggering latency was defined as the time interval between the onset of the gait-transition event and the actual activation of the exoskeleton assistance command. The measured latency included the observation delay introduced by the input window, feature extraction time, BiLSTM inference time, and communication delay between the decoder and the exoskeleton controller.

Across all online trials, valid stance-to-swing transition events were identified offline from synchronized kinematic data and compared with real-time controller outputs. A successful trigger was defined as a valid stance-to-swing transition detection followed by activation of the exoskeleton assistance command within the predefined control window. Non-triggered steps were defined as valid stance-to-swing transitions that were completed through participant self-initiation without robotic assistance when no valid detection occurred. False activations were defined as unintended exoskeleton triggers occurring without corresponding gait-transition events.

Offline and online decoding performance was evaluated using classification accuracy and recall. Accuracy was calculated as the proportion of correctly classified gait states among all predictions:

$$\text{Accuracy}=\frac{\mathrm{TP}+\mathrm{TN}}{\mathrm{TP}+\mathrm{TN}+\mathrm{FP}+\mathrm{FN}}$$

Recall was defined as the proportion of correctly detected stance-to-swing transitions among all actual transitions:

$$\text{Recall}=\frac{\mathrm{TP}}{\mathrm{TP}+\mathrm{FN}}$$

For closed-loop evaluation, successful triggers were defined as valid stance-to-swing intention detections followed by activation of the exoskeleton assistance command within the predefined control window. The successful trigger rate was calculated as the proportion of valid gait-transition intentions that resulted in robotic assistance.

Non-triggered steps were defined as valid stance-to-swing transitions that were not detected by the controller and were subsequently completed through participant self-initiation without robotic assistance.

False activations were defined as unintended exoskeleton assistance commands occurring without a corresponding valid stance-to-swing transition. The total number of false activations was recorded across all online trials.

Task efficiency was evaluated by measuring the total completion time of each walking session under different control conditions. Session time was defined as the elapsed time required to complete all trials within each condition, including walking and turning periods but excluding scheduled rest intervals.

### Gait-cycle segmentation

Gait cycles were defined using hip-joint kinematics captured by the exoskeleton’s embedded sensors. For each stride, the cycle start was marked when the hip angle, following its maximum flexion, decreased to 30° of flexion—signifying the onset of the stance phase. Stance comprised roughly 45% of the total cycle, during which the hip extended from 30° flexion through neutral to its peak extension. The transition to swing was identified at this peak extension, after which the swing phase unfolded as the hip flexed from maximal extension back to its subsequent peak flexion. Finally, a return to 30° flexion closed the cycle and prepared for the next stance onset. Hip kinematics were used solely for offline gait-event labeling and temporal alignment of the detection window; they were not included as input features to the BiLSTM model. The BiLSTM decoder used only EEG–EMG features, and its output probability was evaluated within the predefined stance-to-swing transition window for real-time assistance triggering.

### EEG preprocessing

All 16 EEG channels were retained for analysis. Preprocessing was performed in EEGLAB as follows. First, raw signals were high-pass filtered at 0.1 Hz using a zero-phase, bidirectional FIR filter to remove slow baseline drifts. A subsequent zero-phase FIR low-pass filter at 40 Hz attenuated high-frequency noise, and a notch filter (48–52 Hz) eliminated power-line interference.

Following filtering, any channels exhibiting abnormal activity were identified and interpolated via spherical-spline interpolation based on standard 10–20 electrode locations. We then ran independent component analysis (ICA) to decompose the data into maximally independent sources. Components reflecting eye movements, muscle artifacts, or residual line noise were flagged by visual inspection and removed before reconstructing the clean EEG signals.

Finally, the data were re-referenced to the common average reference to improve spatial resolution and minimize bias from any single electrode. All offline preprocessing parameters were applied uniformly across participants, ensuring consistent data quality for subsequent time-frequency and connectivity analyses.

Offline EEG preprocessing was performed in MATLAB, whereas the online decoding pipeline implemented only causal filtering and real-time feature extraction to satisfy latency requirements. For online control, ICA-based artifact rejection was not applied because the additional computational latency introduced by real-time ICA decomposition was incompatible with the low-latency requirements of exoskeleton triggering. Instead, the online decoder operated on continuously acquired EEG signals after causal filtering and real-time band-power extraction. The potential influence of movement-related artifacts was considered as an important limitation of the present study. The influence of motion-related artifacts was partially mitigated through multimodal fusion, as EEG and EMG information were jointly considered rather than relying exclusively on EEG signals. Although explicit ICA-based artifact rejection was not implemented during online decoding due to real-time computational constraints, several measures were adopted to reduce the influence of non-neural signals. First, the online decoder used only EEG-derived band-power features extracted from predefined sensorimotor-related frequency bands rather than raw EEG amplitudes or exoskeleton kinematic variables. Second, hip-joint kinematics and actuator-related signals were used exclusively for gait-event labeling and synchronization and were not provided as inputs to the BiLSTM classifier. Third, the hybrid model integrated EEG and EMG features within a unified multimodal framework, allowing cortical-related and peripheral neuromuscular information to be jointly represented during gait-event prediction. Nevertheless, the contribution of movement-related EEG artifacts cannot be completely excluded in mobile walking conditions. Potential motion-related contamination was not explicitly removed during online operation; however, the hybrid decoding framework integrated EEG with EMG features, reducing the reliance on EEG signals alone for intention detection.

### EEG power spectral density analysis

To characterize EEG sensorimotor oscillatory dynamics during exoskeleton-assisted gait, we computed power spectral density (PSD) features from EEG data segmented by gait phase. First, EEG recordings were partitioned into support and swing phases for each leg using the gait-cycle boundaries defined by hip-joint kinematics. Within each phase segment, we applied zero-padding to the time-domain signal and performed a Fast Fourier Transform (FFT) via MATLAB’s FFT function. The squared magnitude of the resulting complex spectrum yielded an estimate of instantaneous power across frequencies; these values were then averaged over all time points within the segment to compute a PSD profile.

Power estimates were integrated over four canonical bands— *θ* (4–7 Hz), *μ* (8–13 Hz), low *β* (14–20 Hz), and high *β* (21–30 Hz)—for each EEG channel and gait phase. Finally, PSD values were averaged across all gait cycles to produce representative band-power metrics for the support and swing conditions under each control mode. For ERD analysis, a resting-state EEG baseline was recorded prior to each walking trial while participants maintained a quiet upright standing posture for 60 s. The mean band power calculated from this baseline period was used as the reference value for ERD computation. ERD was calculated as:

$$\mathrm{ERD}(\%)=\frac{\left(P_{\text{baseline}}-P_{\text{phase}}\right)}{P_{\text{baseline}}}*100$$

where P_baseline_ represents the mean band power during the resting baseline period and P_phase_ represents the band power during the corresponding gait phase.

ERD analysis was performed to characterize phase-dependent EEG modulation, whereas the online BiLSTM decoder used band-power features directly as model inputs. Therefore, ERD values were used for neurophysiological interpretation only and were not used as direct triggering criteria in the real-time control system.

### EMG processing

Raw EMG signals from the pilot feasibility study were first band-pass filtered between 20 and 250 Hz using a zero-phase, 4th-order Butterworth filter to remove movement artifacts and high-frequency noise. Each filtered channel was full-wave rectified and envelope-detected to produce smooth amplitude profiles. Using the exoskeleton’s hip-angle trace, we then segmented the envelope signals into individual gait cycles and normalized each cycle to a 0–100% time base, enabling direct comparison across strides.

### Kinematic and latency analysis

Hip-joint kinematic data were imported from CSV logs into MATLAB and time-stamped, with all signals sampled and stored at 100 Hz. During an initial quiet-standing period, hip angles were baseline-corrected to zero, then low-pass filtered at 6 Hz using a fourth-order Butterworth filter to suppress high-frequency noise. Concurrently, EEG was acquired at 500 Hz (transmitted and logged at 100 Hz) via a 16-channel cap, and surface EMG was sampled at 600 Hz; EMG signals underwent envelope detection without further software filtering to yield dynamic activation traces.

To quantify per-step response latency, we defined a ± 1° detection window around the −10° hip-extension setpoint. The filtered hip-angle signal was binarized within this window, and the rising edge of each mask identified the stance-to-swing transition onset time t_i_^peak^. Actuator torque onset times t_i_^assist^ were retrieved from the exoskeleton’s internal log. Latency for each step was calculated as

$$\Delta_{i}=t_{i}^{\text{assist}}-t_{i}^{\text{peak}}$$

To ensure robust estimates, any Δ_i_ falling outside ±2 standard deviations of that trial’s latency distribution was excluded as an outlier.

### BiLSTM model architecture and training

A bidirectional long short-term memory (BiLSTM) network was used for intention detection. The network consisted of two bidirectional LSTM layers followed by a fully connected layer and a SoftMax output. Each LSTM layer contained 64 hidden units. Dropout regularization was applied between layers to reduce overfitting. Input features consisted of time-windowed EEG band power features, EMG envelope features, or their combination in the hybrid mode. EEG inputs consisted of band-power features extracted from the four frequency bands (*θ*, *μ*, low-*β*, and high-*β*) across 16 channels (64 features), whereas EMG inputs consisted of normalized envelope features from six bilateral lower-limb muscles (12 EMG channels/features, representing six muscles from both left and right sides). For the hybrid condition, EEG and EMG features were concatenated into a unified 76-dimensional multimodal feature vector and provided as a single input sequence to the BiLSTM network. EEG and EMG signals were not processed by separate modality-specific detectors; instead, the hybrid decoder used a single pretrained BiLSTM model to integrate both modalities. The model generated a unified classification probability, and a predefined decision threshold was applied to determine whether stance-to-swing assistance was triggered. The hip kinematic signals were used only as external reference signals for offline labeling of gait transitions and were not included in the model input. Therefore, the classifier was trained to predict gait-event-related states from EEG and EMG features rather than directly learning mechanical trajectories. All features were normalized using subject-independent scaling before model training. Temporal windows of 1 s were constructed using a sliding-window approach with overlap to preserve sequential gait-related information while enabling continuous real-time prediction. The sliding window was updated continuously as new samples became available. At each prediction step, the latest available 1-s EEG–EMG feature sequence was processed by the pretrained BiLSTM model to generate a classification probability. Although the model architecture was bidirectional, the backward LSTM branch operated only within the acquired temporal window and did not access future samples beyond the real-time prediction point. Therefore, the backward LSTM branch operated within the available historical window and did not access future samples beyond the real-time prediction point. After segmentation, 9,600 temporal windows were generated for training, 1,200 windows for validation, and 1,200 windows for independent testing. The dataset was split at the subject level to prevent data leakage, with 40 participants used for training, 5 participants used for validation, and 5 participants reserved for independent testing. No participant appeared in more than one subset. The validation set was used for hyperparameter selection and early stopping based on validation loss, whereas the independent test set was used only for final performance evaluation. The model output was a binary classification probability corresponding to stance-to-swing intention. The generated classification probability was compared with a predefined decision threshold to determine whether a valid gait-transition intention was detected and whether exoskeleton assistance was triggered. The decision threshold was determined using the validation dataset during model calibration and was fixed before online experiments without further adjustment. The model was trained using the Adam optimizer with a fixed learning rate. Training was conducted for a predefined number of epochs with early stopping based on validation loss.

## Statistical analysis

All analyses were performed in MATLAB R2024a (MathWorks) and SPSS 28.0 (IBM). Continuous variables—including per-step latency, classification accuracy, trial duration, total session time, donning/doffing times, and QUEST 2.0 scores—were assessed for normality using the Shapiro–Wilk test.

Comparisons among EMG, EEG, and hybrid conditions for latency, accuracy, and total time employed one-way repeated-measures ANOVA; significant main effects were followed by Bonferroni-adjusted pairwise tests. Trial order was additionally examined as a covariate in repeated-measures ANCOVA to evaluate potential practice or fatigue effects. Donning, setup, and doffing times and QUEST 2.0-dimension scores were analyzed with repeated-measures ANOVA when normally distributed; otherwise, the Friedman test with Wilcoxon signed-rank *post hoc* comparisons was used. Effect sizes were reported as partial eta squared (*η*^2^p) for repeated-measures ANOVA/ANCOVA results and Cohen’s d for pairwise comparisons. For continuous outcomes, including triggering latency and completion time, 95% confidence intervals (CIs) were calculated where applicable. All tests were two-tailed with *α* = 0.05. Results are reported as mean ± SD.

## Results

### Dataset overview and trial inclusion

All offline analyses were conducted on the dataset collected from 50 healthy participants. For online experiments, data from seven participants were included. All participants completed the three control conditions (EEG-only, EMG-only, and hybrid EEG–EMG) without adverse events. For performance evaluation, intention detection metrics were computed at the step level and then averaged per subject. All statistical analyses were conducted at the subject level. Only steady-state gait cycles with valid stance-to-swing transitions were included in the analysis. Gait initiation and termination steps were excluded.

### Offline intention detection performance

For the left limb, the hybrid model achieved an accuracy of 91.3% and a recall of 92.5%, outperforming both EEG-only and EMG-only configurations. Similarly, for the right limb, the hybrid mode yielded the highest performance, with an accuracy of 86.6% and a recall of 87.5%.

In contrast, single-modality performance showed greater variability between limbs. While EMG-only control achieved moderate accuracy, its recall on the right limb was substantially lower (62.3%), indicating a higher rate of missed stance-to-swing transitions. EEG-only control demonstrated more balanced recall across limbs but did not reach the performance level of the hybrid configuration.

Subject-level repeated-measures analysis revealed a significant main effect of control mode on both accuracy and recall. For accuracy, repeated-measures ANOVA demonstrated a significant effect of control mode (*F*(2,8) = 12.45, *p* = 0.003, *η*^2^p = 0.76), with post-hoc comparisons confirming the superiority of the hybrid EEG–EMG approach over EEG-only and EMG-only modes. For recall, a significant effect of control mode was also observed (*F*(2,8) = 9.87, *p* = 0.007, *η*^2^p = 0.71). Effect sizes for the hybrid versus single-modality comparisons were in the medium-to-large range, indicating a robust improvement in offline decoding performance (see Table 2).

**Table 2:** Classification performance across control modalities and limbs.

Metric	EEG (left)	EMG (left)	Hybrid (left)	EEG (right)	EMG (right)	Hybrid (right)
ACC	81.8%	81.3%	91.3%	83.5%	81.9%	86.6%
Recall	83.0%	71.9%	92.5%	84.5%	62.3%	87.5%

### Pilot feasibility study

**Figure 4:**
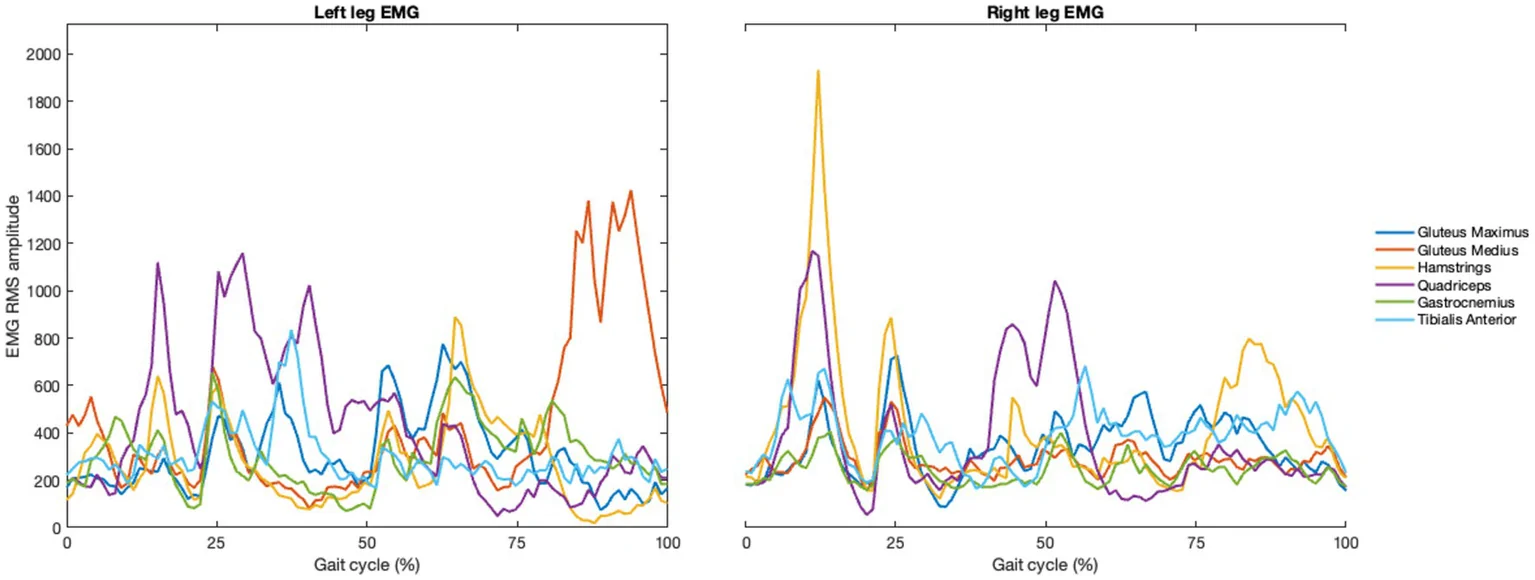
Grand-averaged EMG envelopes across the gait cycle for the left and right lower limbs.

**Figure 5:**
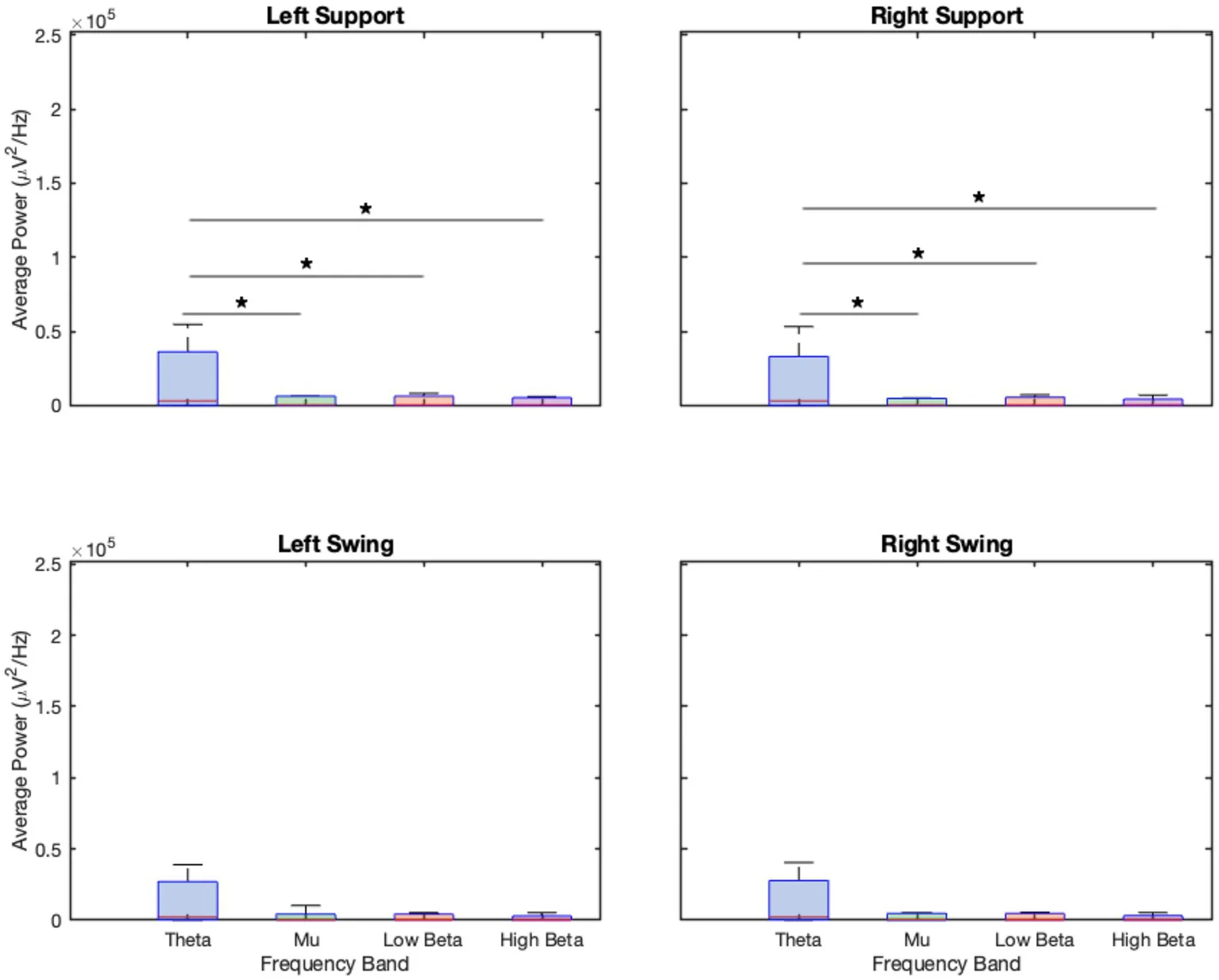
Average power differences calculated across four frequency bands during different gait phases. Results of the post-hoc multiple comparisons between frequency bands are reported (*p* < 0.01).

As illustrated in Figure 4, grand-averaged EMG envelopes across the gait cycle are shown for the left and right lower limbs, with all signals time-normalized to the gait cycle (0%–100%). Each panel depicts the mean activation profiles of six lower-limb muscles, including the gluteus maximus, gluteus medius, hamstrings, quadriceps femoris, gastrocnemius, and tibialis anterior. Across muscles, clear phase-dependent activation patterns were observed, characterized by distinct bursts occurring at consistent portions of the gait cycle. Proximal muscles such as the gluteus maximus and gluteus medius exhibited increased activation during the early stance phase, whereas the quadriceps femoris showed prominent activity during weight acceptance. In contrast, the hamstrings and tibialis anterior demonstrated heightened activation toward late stance and swing-related phases, reflecting their roles in limb deceleration and foot clearance. Importantly, the overall temporal structure of muscle activation was highly consistent between the left and right limbs, with comparable peak locations and envelope shapes across the gait cycle. While minor amplitude differences were present between sides, the relative timing of muscle recruitment remained stable, indicating preserved inter-limb coordination. The smooth and repeatable EMG envelopes further confirm the robustness of the preprocessing and gait-cycle segmentation procedures, supporting their suitability for downstream neuromuscular analysis and hybrid EEG–EMG–based gait decoding (Figure 5).

Average EEG band power was analyzed across four frequency bands (*θ*: 4–7 Hz; *μ*: 8–13 Hz; low *β*: 14–20 Hz; high *β*: 21–30 Hz) during pilot walking trials under the hybrid EEG–EMG control paradigm. Band power distributions are summarized using boxplots for each gait phase, with support phases shown in the top row and swing phases in the bottom row. During the support phase, both the left and right legs exhibited a clear predominance of *θ*-band activity. Median *θ*-band power was significantly higher than *μ*, low *β*, and high *β* bands (*p* < 0.01), whereas power in the *μ* and *β* ranges remained comparatively low. This frequency-specific distribution was consistent across the left and right support conditions, indicating a stable low-frequency cortical engagement pattern during stance. In the swing phase, *θ*-band power remained the dominant component for both legs, although overall spectral power was reduced compared with the support phase. No significant differences were observed among *μ*, low *β*, and high *β* bands during swing, and the distributions were highly similar between the left and right legs. Together, these results demonstrate a consistent *θ*-dominant spectral profile across gait phases under hybrid control, with clear phase-dependent modulation of overall EEG power.

### Online validation

Across all online trials, 420 valid transitions were included for analysis. Among these transitions, 95% of stance-to-swing transition detections were successfully converted into exoskeleton assistance triggers, whereas the remaining non-triggered steps were completed through self-initiation without robotic assistance when no valid detection occurred. False activations were defined as unintended exoskeleton triggers occurring without corresponding gait-transition event, and 20 false activations were observed throughout the online experiments.

### Closed-loop control phase: comparison of EMG, EEG, and hybrid EEG–EMG conditions

**Figure 6:**
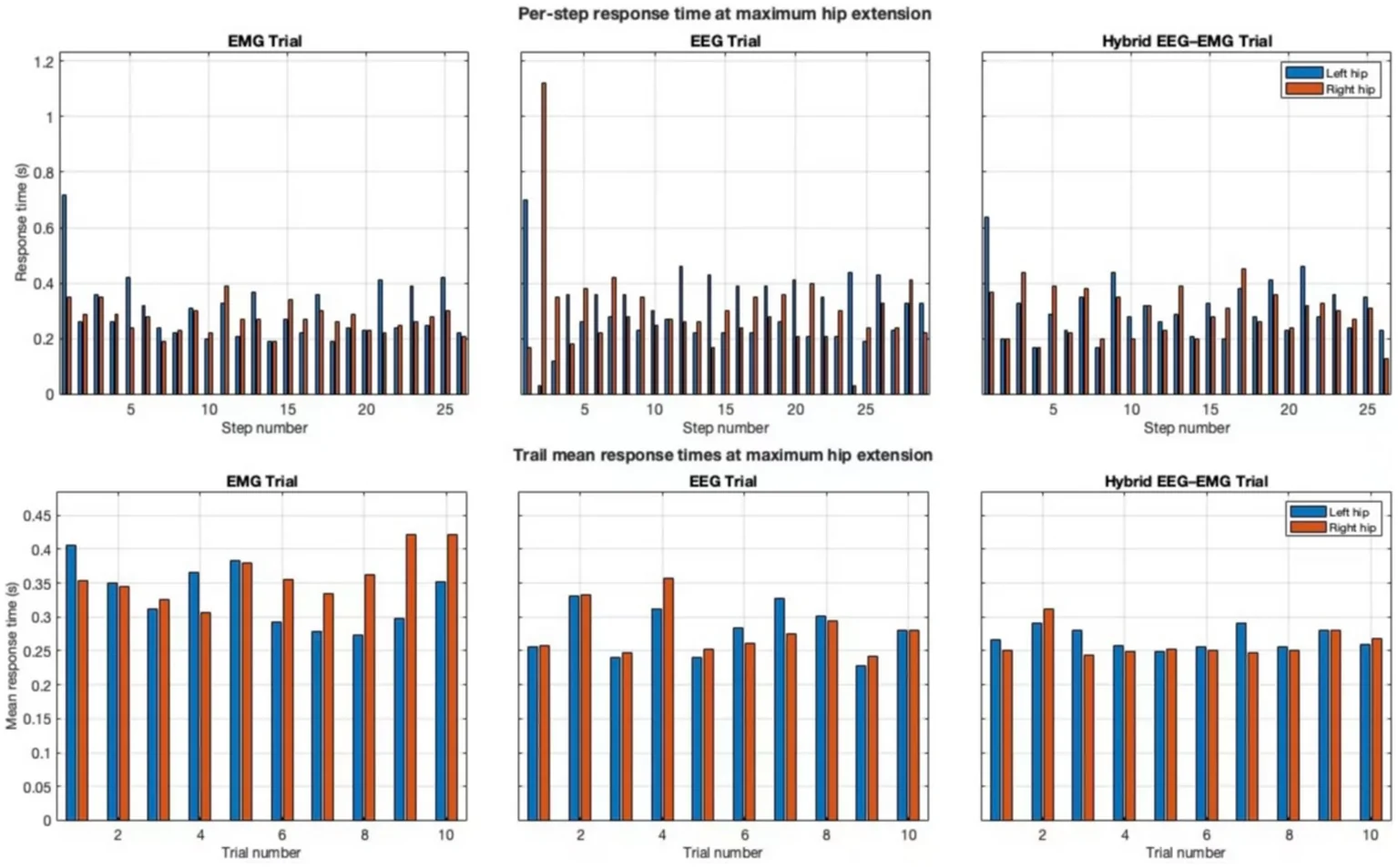
Response time analyses across control conditions.

Per-step response times at maximum hip extension, as shown in Figure 6, reveal clear differences across EMG, EEG, and hybrid EEG–EMG control conditions. Under EMG control, response times vary widely from step to step, typically spanning approximately 0.15–0.45 s. Substantial fluctuations are observed across successive steps, and noticeable left–right asymmetry appears in multiple instances, indicating limited temporal stability of the EMG-only triggering mechanism. EEG-based control exhibits more temporally regular per-step response times. Although variability across steps remains, most responses cluster within a narrower range (approximately 0.20–0.40 s), and bilateral differences are reduced relative to the EMG condition. This pattern suggests that EEG-derived neural markers provide a more consistent trigger timing than muscle activation alone. The hybrid EEG–EMG condition yields the shortest and most stable per-step response times. As illustrated in Figure 6, most steps show latencies below 0.30 s, with visibly reduced dispersion across both hips. Step-to-step variability is markedly attenuated, reflecting improved robustness when cortical and muscular information are combined. These step-level trends are mirrored in the trial-averaged response times. Mean response latencies decrease progressively from EMG to EEG and hybrid control, with EMG trials showing the largest inter-trial variability, EEG trials displaying intermediate dispersion, and hybrid EEG–EMG trials exhibiting the tightest clustering. Across all trials, the average response time (mean ± SD) is 0.33 ± 0.07 s for EMG control, 0.30 ± 0.05 s for EEG control, and 0.28 ± 0.04 s for hybrid EEG–EMG control. Together, the results summarized in Figure 6 demonstrate that multimodal EEG–EMG control enables faster and more consistent real-time assistance triggering at maximum hip extension compared with unimodal strategies.

The per-trial response latencies for each participant under EMG, EEG, and hybrid EEG–EMG control are detailed in Table 3. For each of the ten 20 m trials, left and right hip latencies (mean ± SD) are reported, along with the overall average across all trials (*n* = 10).

**Table 3:** The per-trial response latencies for each participant under EMG, EEG, and hybrid EEG–EMG control.

Participant	Trial	EMG left hip (s)	EMG right hip (s)	EEG left hip (s)	EEG right hip (s)	HybridLeft hip (s)	HybridRight hip (s)
1	1	0.350 ± 0.160	0.323 ± 0.200	0.328 ± 0.143	0.367 ± 0.183	0.375 ± 0.139	0.322 ± 0.088
	2	0.311 ± 0.116	0.318 ± 0.125	0.302 ± 0.114	0.272 ± 0.050	0.372 ± 0.120	0.330 ± 0.150
	3	0.365 ± 0.148	0.318 ± 0.271	0.310 ± 0.126	0.303 ± 0.179	0.301 ± 0.152	0.314 ± 0.139
	4	0.383 ± 0.140	0.357 ± 0.218	0.305 ± 0.104	0.293 ± 0.085	0.311 ± 0.162	0.290 ± 0.107
	5	0.281 ± 0.129	0.355 ± 0.078	0.363 ± 0.131	0.344 ± 0.090	0.283 ± 0.115	0.289 ± 0.084
	6	0.278 ± 0.110	0.335 ± 0.093	0.361 ± 0.132	0.336 ± 0.108	0.306 ± 0.177	0.298 ± 0.124
	7	0.267 ± 0.119	0.363 ± 0.151	0.357 ± 0.144	0.303 ± 0.055	0.322 ± 0.154	0.286 ± 0.094
	8	0.298 ± 0.075	0.421 ± 0.106	0.352 ± 0.129	0.311 ± 0.059	0.311 ± 0.100	0.305 ± 0.096
	9	0.353 ± 0.121	0.428 ± 0.189	0.353 ± 0.136	0.340 ± 0.127	0.284 ± 0.117	0.297 ± 0.079
	10	0.407 ± 0.145	0.365 ± 0.229	0.348 ± 0.140	0.303 ± 0.079	0.291 ± 0.112	0.293 ± 0.067
	Overall (*n* = 10)	0.331 ± 0.047	0.361 ± 0.038	0.338 ± 0.025	0.318 ± 0.029	0.316 ± 0.034	0.302 ± 0.015
2	1	0.358 ± 0.187	0.338 ± 0.147	0.361 ± 0.131	0.378 ± 0.192	0.275 ± 0.174	0.322 ± 0.122
	2	0.448 ± 0.182	0.457 ± 0.157	0.300 ± 0.146	0.347 ± 0.111	0.414 ± 0.139	0.376 ± 0.122
	3	0.338 ± 0.134	0.321 ± 0.106	0.371 ± 0.129	0.393 ± 0.211	0.349 ± 0.136	0.354 ± 0.090
	4	0.427 ± 0.158	0.497 ± 0.137	0.340 ± 0.155	0.378 ± 0.196	0.309 ± 0.091	0.315 ± 0.101
	5	0.338 ± 0.175	0.328 ± 0.133	0.348 ± 0.108	0.404 ± 0.179	0.267 ± 0.102	0.264 ± 0.081
	6	0.391 ± 0.179	0.344 ± 0.181	0.318 ± 0.139	0.365 ± 0.199	0.258 ± 0.127	0.255 ± 0.089
	7	0.446 ± 0.182	0.366 ± 0.138	0.350 ± 0.118	0.392 ± 0.186	0.324 ± 0.123	0.305 ± 0.072
	8	0.413 ± 0.166	0.395 ± 0.156	0.338 ± 0.124	0.290 ± 0.201	0.325 ± 0.233	0.304 ± 0.113
	9	0.323 ± 0.150	0.313 ± 0.115	0.343 ± 0.134	0.392 ± 0.206	0.304 ± 0.116	0.297 ± 0.114
	10	0.341 ± 0.210	0.363 ± 0.131	0.332 ± 0.153	0.359 ± 0.203	0.322 ± 0.121	0.273 ± 0.106
	Overall (*n* = 10)	0.387 ± 0.049	0.373 ± 0.064	0.340 ± 0.020	0.370 ± 0.033	0.315 ± 0.046	0.306 ± 0.038
3	1	0.319 ± 0.142	0.292 ± 0.077	0.327 ± 0.122	0.302 ± 0.107	0.335 ± 0.122	0.345 ± 0.108
	2	0.282 ± 0.080	0.282 ± 0.104	0.344 ± 0.224	0.340 ± 0.126	0.284 ± 0.091	0.270 ± 0.075
	3	0.278 ± 0.155	0.287 ± 0.123	0.320 ± 0.108	0.323 ± 0.080	0.288 ± 0.110	0.277 ± 0.087
	4	0.387 ± 0.198	0.294 ± 0.093	0.290 ± 0.063	0.315 ± 0.134	0.350 ± 0.129	0.314 ± 0.109
	5	0.405 ± 0.195	0.322 ± 0.048	0.296 ± 0.113	0.301 ± 0.108	0.325 ± 0.117	0.290 ± 0.085
	6	0.413 ± 0.169	0.317 ± 0.099	0.255 ± 0.129	0.270 ± 0.090	0.277 ± 0.080	0.262 ± 0.078
	7	0.393 ± 0.162	0.293 ± 0.110	0.318 ± 0.135	0.314 ± 0.070	0.304 ± 0.153	0.280 ± 0.093
	8	0.397 ± 0.207	0.339 ± 0.105	0.264 ± 0.111	0.271 ± 0.074	0.265 ± 0.194	0.253 ± 0.091
	9	0.359 ± 0.166	0.287 ± 0.105	0.279 ± 0.085	0.320 ± 0.078	0.298 ± 0.095	0.316 ± 0.082
	10	0.410 ± 0.144	0.312 ± 0.099	0.303 ± 0.104	0.310 ± 0.065	0.277 ± 0.110	0.288 ± 0.070
	Overall (*n* = 10)	0.364 ± 0.052	0.302 ± 0.019	0.301 ± 0.025	0.312 ± 0.031	0.300 ± 0.028	0.290 ± 0.028
4	1	0.321 ± 0.136	0.277 ± 0.079	0.296 ± 0.179	0.252 ± 0.068	0.267 ± 0.092	0.262 ± 0.098
	2	0.300 ± 0.138	0.313 ± 0.110	0.295 ± 0.120	0.311 ± 0.087	0.292 ± 0.124	0.300 ± 0.100
	3	0.268 ± 0.130	0.289 ± 0.080	0.297 ± 0.126	0.243 ± 0.072	0.280 ± 0.088	0.261 ± 0.092
	4	0.258 ± 0.140	0.300 ± 0.120	0.315 ± 0.158	0.249 ± 0.075	0.258 ± 0.099	0.273 ± 0.081
	5	0.225 ± 0.130	0.266 ± 0.096	0.289 ± 0.130	0.253 ± 0.055	0.248 ± 0.130	0.259 ± 0.090
	6	0.298 ± 0.121	0.278 ± 0.091	0.312 ± 0.189	0.252 ± 0.088	0.256 ± 0.078	0.260 ± 0.076
	7	0.261 ± 0.114	0.293 ± 0.098	0.321 ± 0.215	0.247 ± 0.083	0.292 ± 0.128	0.263 ± 0.064
	8	0.254 ± 0.113	0.277 ± 0.104	0.225 ± 0.072	0.250 ± 0.072	0.256 ± 0.085	0.243 ± 0.107
	9	0.261 ± 0.125	0.272 ± 0.099	0.258 ± 0.120	0.280 ± 0.075	0.280 ± 0.123	0.256 ± 0.098
	10	0.265 ± 0.145	0.263 ± 0.112	0.254 ± 0.097	0.268 ± 0.065	0.259 ± 0.095	0.264 ± 0.086
	Overall (*n* = 10)	0.271 ± 0.028	0.283 ± 0.016	0.286 ± 0.031	0.261 ± 0.021	0.269 ± 0.016	0.264 ± 0.015
5	1	0.337 ± 0.189	0.312 ± 0.114	0.354 ± 0.168	0.323 ± 0.080	0.325 ± 0.114	0.312 ± 0.103
	2	0.323 ± 0.156	0.349 ± 0.130	0.351 ± 0.245	0.294 ± 0.068	0.312 ± 0.145	0.306 ± 0.103
	3	0.298 ± 0.126	0.358 ± 0.098	0.407 ± 0.177	0.321 ± 0.092	0.323 ± 0.144	0.258 ± 0.115
	4	0.328 ± 0.121	0.314 ± 0.115	0.332 ± 0.123	0.305 ± 0.067	0.320 ± 0.158	0.303 ± 0.184
	5	0.303 ± 0.106	0.334 ± 0.092	0.333 ± 0.197	0.301 ± 0.066	0.325 ± 0.114	0.303 ± 0.128
	6	0.324 ± 0.118	0.344 ± 0.086	0.364 ± 0.141	0.281 ± 0.064	0.297 ± 0.134	0.274 ± 0.069
	7	0.283 ± 0.157	0.387 ± 0.199	0.321 ± 0.136	0.292 ± 0.071	0.307 ± 0.126	0.305 ± 0.092
	8	0.447 ± 0.170	0.289 ± 0.078	0.417 ± 0.227	0.297 ± 0.074	0.286 ± 0.114	0.298 ± 0.096
	9	0.261 ± 0.108	0.251 ± 0.077	0.294 ± 0.135	0.328 ± 0.096	0.312 ± 0.097	0.276 ± 0.067
	10	0.353 ± 0.248	0.277 ± 0.044	0.283 ± 0.084	0.301 ± 0.067	0.307 ± 0.120	0.287 ± 0.082
	Overall (*n* = 10)	0.326 ± 0.050	0.321 ± 0.041	0.346 ± 0.043	0.304 ± 0.015	0.312 ± 0.013	0.292 ± 0.018
6	1	0.257 ± 0.077	0.237 ± 0.061	0.293 ± 0.110	0.288 ± 0.067	0.253 ± 0.124	0.249 ± 0.067
	2	0.262 ± 0.104	0.250 ± 0.074	0.294 ± 0.106	0.284 ± 0.058	0.277 ± 0.107	0.258 ± 0.057
	3	0.289 ± 0.122	0.255 ± 0.071	0.288 ± 0.132	0.289 ± 0.062	0.264 ± 0.140	0.261 ± 0.073
	4	0.255 ± 0.094	0.259 ± 0.059	0.285 ± 0.150	0.277 ± 0.067	0.251 ± 0.084	0.234 ± 0.060
	5	0.274 ± 0.096	0.240 ± 0.046	0.280 ± 0.132	0.285 ± 0.068	0.258 ± 0.089	0.262 ± 0.068
	6	0.271 ± 0.092	0.254 ± 0.053	0.304 ± 0.116	0.288 ± 0.081	0.242 ± 0.090	0.254 ± 0.057
	7	0.270 ± 0.111	0.259 ± 0.082	0.322 ± 0.183	0.305 ± 0.081	0.259 ± 0.105	0.241 ± 0.056
	8	0.272 ± 0.105	0.244 ± 0.081	0.332 ± 0.141	0.285 ± 0.068	0.258 ± 0.117	0.259 ± 0.084
	9	0.260 ± 0.102	0.248 ± 0.063	0.331 ± 0.123	0.305 ± 0.045	0.274 ± 0.131	0.250 ± 0.065
	10	0.256 ± 0.113	0.260 ± 0.045	0.284 ± 0.114	0.279 ± 0.050	0.230 ± 0.076	0.247 ± 0.055
	Overall (*n* = 10)	0.267 ± 0.011	0.251 ± 0.008	0.301 ± 0.020	0.288 ± 0.009	0.257 ± 0.014	0.251 ± 0.009
7	1	0.270 ± 0.107	0.248 ± 0.064	0.249 ± 0.091	0.240 ± 0.060	0.267 ± 0.112	0.239 ± 0.068
	2	0.277 ± 0.140	0.303 ± 0.112	0.298 ± 0.093	0.292 ± 0.077	0.325 ± 0.111	0.240 ± 0.070
	3	0.262 ± 0.111	0.272 ± 0.087	0.276 ± 0.106	0.263 ± 0.073	0.253 ± 0.091	0.244 ± 0.052
	4	0.249 ± 0.104	0.267 ± 0.101	0.278 ± 0.112	0.278 ± 0.085	0.264 ± 0.110	0.239 ± 0.061
	5	0.286 ± 0.134	0.248 ± 0.099	0.427 ± 0.279	0.293 ± 0.176	0.261 ± 0.114	0.241 ± 0.058
	6	0.293 ± 0.109	0.256 ± 0.082	0.269 ± 0.063	0.251 ± 0.067	0.288 ± 0.142	0.241 ± 0.056
	7	0.300 ± 0.131	0.278 ± 0.074	0.241 ± 0.075	0.233 ± 0.062	0.258 ± 0.102	0.249 ± 0.049
	8	0.299 ± 0.112	0.275 ± 0.082	0.260 ± 0.115	0.257 ± 0.065	0.275 ± 0.098	0.247 ± 0.067
	9	0.266 ± 0.099	0.259 ± 0.067	0.246 ± 0.087	0.244 ± 0.061	0.281 ± 0.145	0.245 ± 0.060
	10	0.257 ± 0.120	0.271 ± 0.075	0.248 ± 0.088	0.256 ± 0.070	0.256 ± 0.097	0.241 ± 0.087
	Overall (*n* = 10)	0.276 ± 0.018	0.268 ± 0.016	0.279 ± 0.055	0.261 ± 0.021	0.273 ± 0.021	0.243 ± 0.004

**Figure 7:**
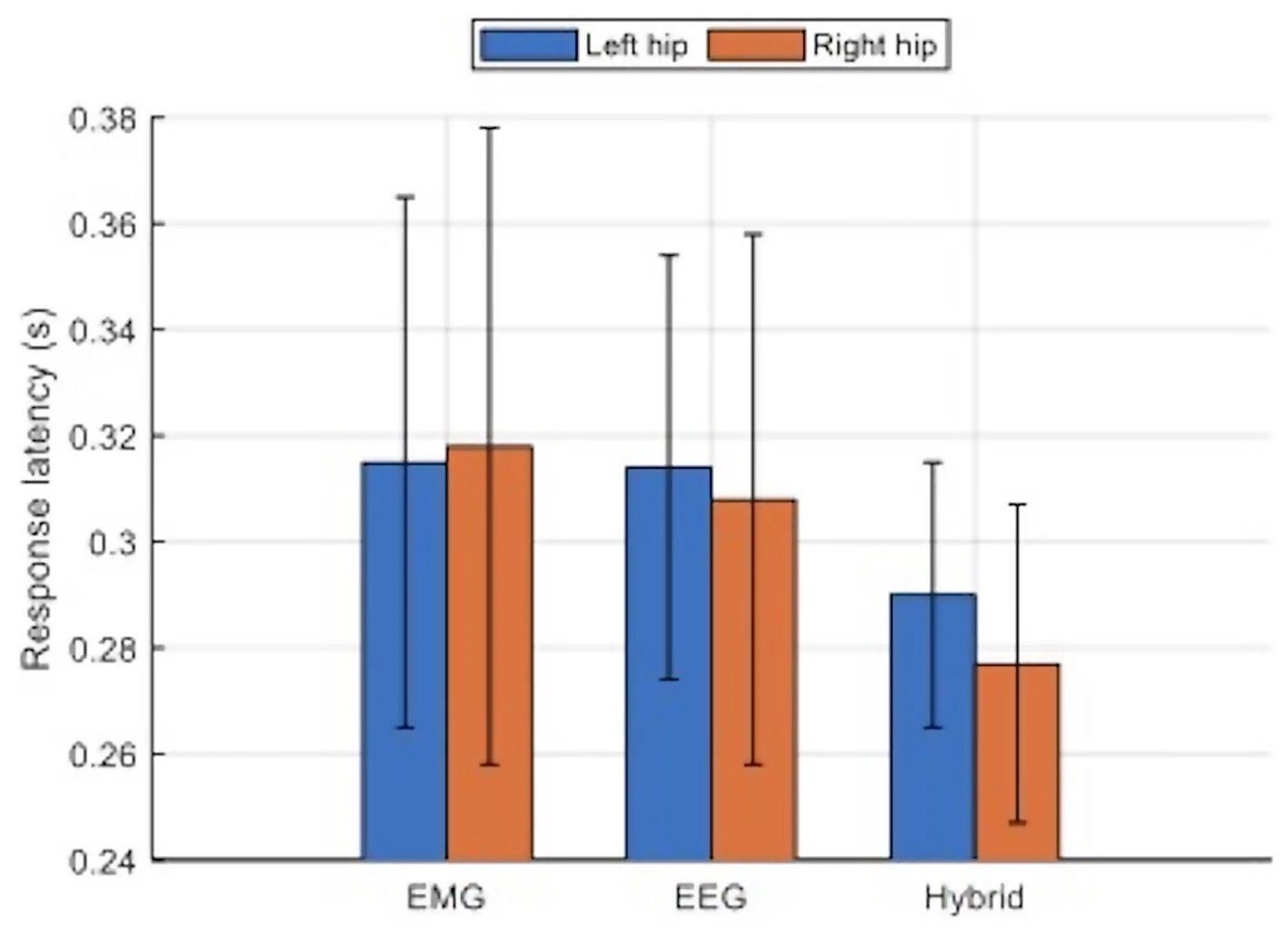
Mean step response latencies for left and right hip across EMG, EEG, and hybrid control conditions.

Across ten trials and seven participants, step-level response latencies differed systematically among EMG, EEG, and hybrid EEG–EMG control modes, with detailed values summarized in Table 3. When averaged across subjects, EMG-based control exhibited the longest response times and the greatest inter-subject variability (left hip: 0.316 ± 0.055 s; right hip: 0.318 ± 0.059 s). EEG control was associated with reduced dispersion and moderately lower mean latencies (left: 0.316 ± 0.039 s; right: 0.309 ± 0.053 s), indicating more consistent temporal alignment of assistance onset. The hybrid EEG–EMG paradigm yielded the shortest and most stable response times across participants (left: 0.291 ± 0.025 s; right: 0.277 ± 0.029 s). Compared with EMG-only control, hybrid control significantly reduced response latency by 33 ms (95% CI: 18–66 ms, *p* = 0.012, Cohen’s d = 0.88). These subject-level patterns are consistent with the aggregate trends illustrated in Figure 7, demonstrating improved responsiveness with multimodal control compared with unimodal strategies.

Per-trial response durations for each of the seven subjects across ten 20-m walking trials are listed in Tables 4–6 for the EMG, EEG, and hybrid EEG–EMG control conditions, respectively. All durations were extracted directly from the exoskeleton’s internal log files, calculated from timestamped trial onset and offset markers, and resampled to a unified temporal resolution of 100 Hz. For each subject, total session time was obtained by summing the durations of the ten trials and subsequently converting the cumulative values to minutes.

**Table 4:** EMG mode: per-trial and total durations.

Subject	Trial 1 (s)	Trial 2 (s)	Trial 3 (s)	Trial 4 (s)	Trial 5 (s)	Trial 6 (s)	Trial 7 (s)	Trial 8 (s)	Trial 9 (s)	Trial 10 (s)
1	184.93	154.88	185.60	192.32	213.16	172.76	198.65	178.91	176.01	178.08
2	184.55	201.67	181.60	188.50	170.52	175.73	180.98	171.41	358.33	246.91
3	248.44	349.09	214.30	215.68	202.43	211.99	216.74	219.21	202.48	211.27
4	235.79	274.27	282.37	270.83	248.66	255.51	235.25	227.81	223.87	222.43
5	177.30	181.25	219.33	204.95	197.10	214.57	193.76	187.07	203.83	280.65
6	202.67	181.92	223.41	178.46	190.62	185.31	191.26	176.83	192.45	184.16
7	211.02	186.77	206.98	201.68	209.62	182.90	195.38	194.04	219.57	183.40

**Table 5:** EEG mode: per-trial and total durations.

Subject	Trial 1 (s)	Trial 2 (s)	Trial 3 (s)	Trial 4 (s)	Trial 5 (s)	Trial 6 (s)	Trial 7 (s)	Trial 8 (s)	Trial 9 (s)	Trial 10 (s)
1	162.10	223.30	227.06	161.03	224.12	194.92	194.81	182.53	197.64	209.85
2	186.98	251.18	304.88	276.32	207.73	282.85	234.79	239.69	274.75	231.56
3	191.51	216.11	210.89	192.27	216.97	211.20	209.64	217.57	209.57	213.32
4	271.10	257.12	250.17	234.16	230.53	231.37	235.40	227.72	246.59	224.18
5	216.52	210.98	211.30	199.51	206.18	217.97	215.91	194.22	217.46	225.32
6	195.17	260.26	197.46	197.01	190.95	185.19	184.38	176.12	173.44	183.43
7	204.25	197.61	171.87	169.93	187.68	194.60	201.92	202.68	211.21	223.25

**Table 6:** Hybrid mode: per-trial and total durations.

Subject	Trial 1 (s)	Trial 2 (s)	Trial 3 (s)	Trial 4 (s)	Trial 5 (s)	Trial 6 (s)	Trial 7 (s)	Trial 8 (s)	Trial 9 (s)	Trial 10 (s)
1	171.93	190.02	185.24	176.94	178.10	181.60	178.77	181.16	202.28	182.85
2	185.39	166.79	165.28	203.87	187.68	191.50	187.27	185.26	191.69	188.09
3	218.73	212.64	217.55	186.55	208.57	171.50	205.82	207.26	214.47	208.39
4	183.57	223.58	207.23	208.03	226.74	219.21	200.41	206.72	276.25	207.99
5	190.94	222.28	179.19	216.31	195.39	193.99	192.92	175.17	201.93	168.27
6	189.68	219.99	188.83	187.39	186.45	183.56	188.27	190.35	186.53	174.24
7	217.89	173.86	172.97	164.07	177.50	164.87	168.22	172.19	172.21	173.08

**Figure 8:**
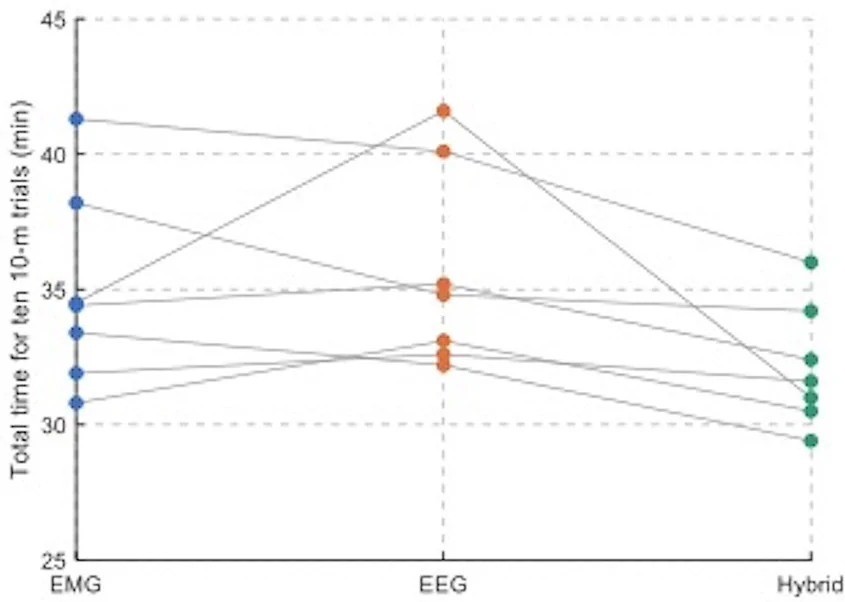
Task completion time across control modes.

Figure 8 presents a paired, subject-level comparison of the total duration required to complete ten 20-m walking trials (10 m outward and 10 m return) under the three control modes. For each participant, total task duration was computed by summing the completion times of all ten trials, and comparisons were conducted within subjects across EMG-only, EEG-only, and hybrid EEG–EMG conditions.

As illustrated by the paired line and dot representation, the hybrid control mode consistently resulted in shorter task completion times for most participants. Most subject-specific trajectories showed a decrease from EMG-only or EEG-only control to hybrid control, indicating that the observed improvement was not driven by a small subset of participants but reflected a systematic within-subject effect.

Across subjects, EMG-only and EEG-only modes exhibited greater inter-individual variability in total duration, whereas the hybrid mode demonstrated both reduced completion time and reduced dispersion. This trend is consistent with the improved decoding accuracy and reduced response latency observed under hybrid control, suggesting that more reliable gait-transition detection translated into improved functional task efficiency.

Importantly, results were analyzed at the participant level rather than pooled across individual steps, thereby avoiding violations of independence assumptions. Statistical comparisons were therefore performed using participant-level mean values rather than individual step-level observations.

Across participants, hybrid EEG–EMG control resulted in the shortest total completion time (32.3 ± 2.4 min) compared with EMG-only (35.0 ± 3.8 min) and EEG-only (35.7 ± 3.6 min) conditions. A repeated-measures ANOVA revealed a significant main effect of control mode (*F*(2,12) = 6.84, *p* = 0.010, *η*^2^p = 0.533). Bonferroni-adjusted *post hoc* comparisons showed that hybrid control significantly reduced total task duration compared with EMG-only control (mean difference: −2.7 min, 95% CI [−4.8, −0.6], *p* = 0.018, Cohen’s d = 0.91) and EEG-only control (mean difference: −3.4 min, 95% CI [−5.5, −1.3], *p* = 0.009, Cohen’s d = 1.05).

Across seven able-bodied participants, the time required for donning, system setup, and doffing the wearable lower-limb exoskeleton is detailed in Table 7. Donning showed the greatest inter-individual variability, with durations ranging from 8.23 to 26.88 min (mean ± SD: 13.28 ± 6.56 min). In contrast, system setup—including initialization and signal verification—was considerably shorter and more consistent across participants, averaging 3.40 ± 1.73 min (range: 0.78–5.30 min). Doffing required a comparable amount of time, with a mean duration of 3.00 ± 0.90 min (range: 1.77–4.55 min).

**Table 7:** Time to don, set up, and doff the wearable exoskeleton.

Subject	Donning (min)	Setup (min)	Doffing (min)
1	26.88	1.25	4.55
2	15.60	0.78	3.42
3	13.30	5.30	1.77
4	10.75	4.92	3.28
5	8.33	3.92	2.58
6	8.23	4.02	2.27
7	9.87	3.62	3.12
Mean ± SD	13.28 ± 6.56	3.40 ± 1.73	3.00 ± 0.90

**Figure 9:**
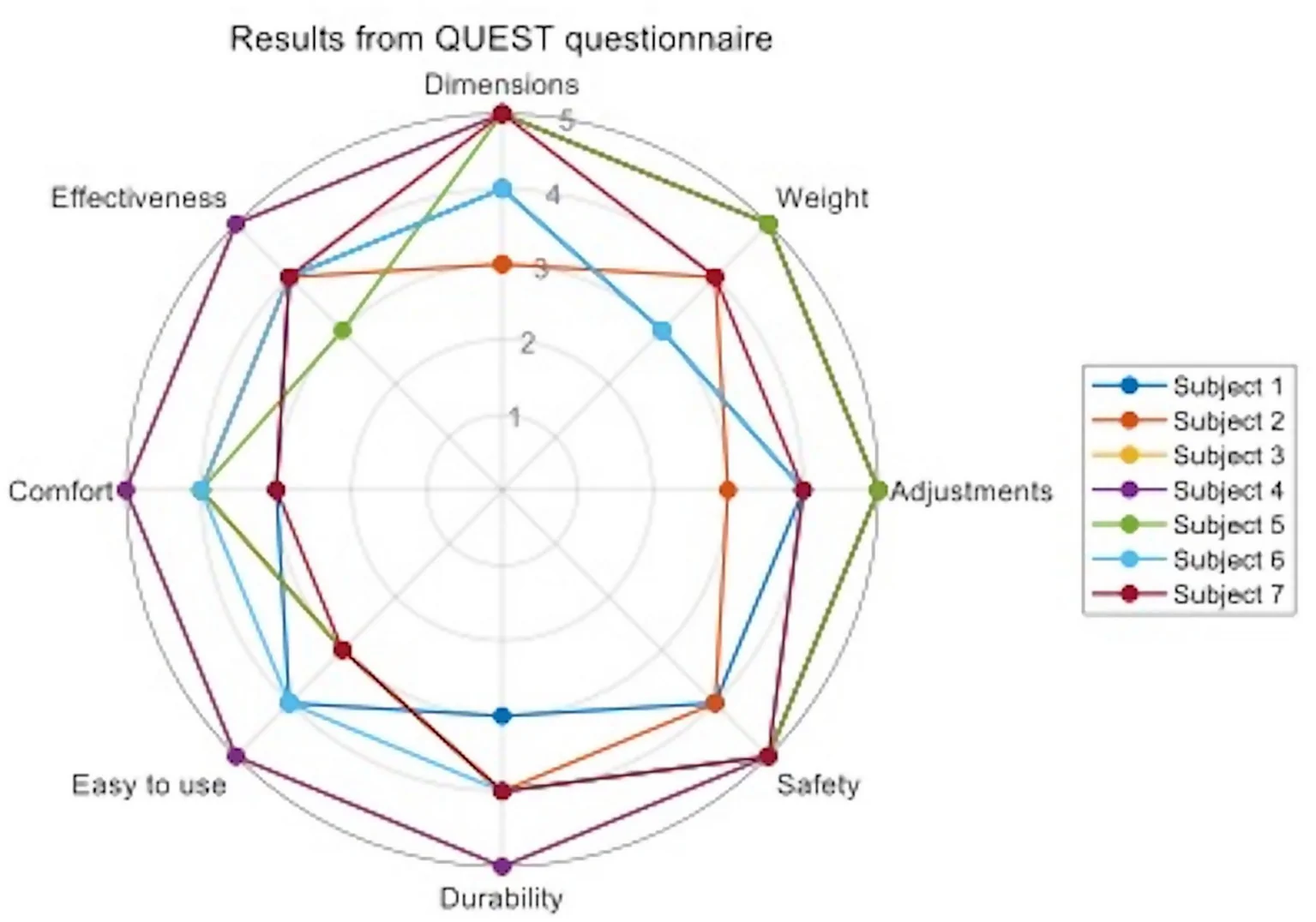
QUEST 2.0 scores for device satisfaction items.

The radar plot (see Figure 9) displays each of the seven participants’ QUEST 2.0 scores for device satisfaction, spanning Dimensions, Weight, Adjustments, Safety, Durability, Ease of Use, Comfort, and Effectiveness. Each colored line corresponds to one subject’s mean rating on a 0–5 scale, with the concentric circles marking integer score levels. All subjects rated Safety and Effectiveness highly (mean scores ≥4.5), indicating robust confidence in the exoskeleton’s protective features and functional assistance. Durability and Adjustments also received consistently strong ratings (means ≥4.2), reflecting ease of fit and perceived reliability during use. In contrast, Comfort and Weight showed greater variability (means of 3.7 and 3.9, SDs of 0.6 and 0.7, respectively), suggesting individual differences in subjective perception of bulk and wearability. Ease of Use and Dimensions occupied intermediate positions (means ≈4.0), demonstrating generally favorable but varied experiences with donning procedures and device sizing.

## Discussion

We developed and validated a real-time BiLSTM-based hybrid EEG–EMG intention detection framework and tested it in seven able-bodied participants. Across offline and online evaluations, the hybrid configuration consistently outperformed single-modality approaches: it achieved the highest classification accuracy (Left: 91.3%; Right: 86.6%) and recall (Left: 92.5%; Right: 87.5%), and the shortest mean per-step latencies (hybrid Left ≈ 0.29 s, Right ≈ 0.28 s) compared with EMG-only and EEG-only modes (Results; Table 2, Figure 6). The latency difference between EMG and hybrid control corresponded to a large effect size (Cohen’s d ≈ 0.88). Usability measures (donning/setup/doffing and QUEST 2.0) indicate acceptable operational burden for a laboratory proof-of-concept.

### Model validation

Our comparative evaluation demonstrates that the BiLSTM-based hybrid EEG–EMG model substantially outperforms unimodal classifiers in predicting critical gait events-such as stance-to-swing transitions and peak hip flexion moments and peak hip flexion moments-based on hip-angle segmentation (Tables 2, 3). The hybrid approach attained accuracies of 91.3% (left) and 86.6% (right), exceeding the EEG-only model by ~9%–10% and the EMG-only model by 5%–10%. This performance gain likely derives from complementary information provided by the two modalities: EEG-derived sensorimotor rhythm features may provide information associated with gait-intention-related transitions, whereas EMG envelope bursts provide temporally precise information related to peripheral muscle activation. The bidirectional temporal processing inherent to the BiLSTM architecture enables effective capture of these time-dependent neuromuscular features across gait cycles, leveraging past and future context for robust phase classification (Graves and Schmidhuber, 2005).

Sensitivity (recall) further underscores modality-specific trade-offs. EEG detection achieved 83.0% (left) and 84.5% (right) recall-missing roughly one in six true transitions, likely due to cortical signal attenuation and decoding latency-whereas EMG recall was markedly lower (71.9% left; 62.3% right), reflecting variability in lower-limb muscle recruitment and sensor placement. The hybrid model’s recall of 92.5% (left) and 87.5% (right) not only boosted sensitivity but also narrowed the inter-limb gap to 5.0%, ensuring balanced bilateral assistance critical for stable gait. High overall accuracy further indicates minimized false positives, preserving system responsiveness without spurious triggers.

We observed inter-limb differences in some unimodal results (notably lower EMG recall on the right limb, 62.3%) and mild left–right asymmetry in latencies. Several non-exclusive explanations are plausible: (1) sensor placement variability or differential skin–electrode contact; (2) intrinsic inter-limb dominance or habitual asymmetries in motor control (all participants were right-handed); (3) subtle biomechanical differences during assisted walking (e.g., small differences in assistive linkage or fit); and (4) remaining EMG crosstalk or low SNR for particular muscles. Importantly, the hybrid model substantially reduced the inter-limb gap (hybrid recall gap ≈5%), suggesting multimodal fusion mitigates modality-specific weaknesses. We therefore recommend that future iterations include systematic per-muscle signal quality checks, ankle/foot sensors for independent kinematic references, and—where feasible—standardized electrode positioning protocols to further reduce asymmetry.

These findings corroborate the presence of complementary information in cortical and muscular signals and validate multimodal fusion as a pathway to heightened classification reliability under diverse gait dynamics (Garro et al., 2021),they align with earlier Bayesian fusion of EEG and EMG classifiers (Leeb et al., 2011), contrastive training paradigms that leverage cross-modal supervision (De Seta and Romeni, 2025), and emerging AI frameworks handling high-dimensional biomechanical data (Pan et al., 2026). Moreover, the hybrid model’s robustness across subjects and gait tasks addresses known limitations of unimodal decoders in cross-subject generalizability, underscoring its promise for clinical deployment in assistive exoskeleton control. Collectively, these results advance our understanding of neuromuscular coordination during assisted locomotion and pave the way for optimized strategies and real-world brain–machine interface applications.

### Pilot feasibility study

Our combined EEG and EMG analyses suggest that the hybrid system captures intention-related neuromuscular patterns during powered assistance. EMG data show that, when the exoskeleton drives the hip to peak extension, proximal extensors (gluteus maximus/medius) remain tonically active throughout stance, peaking just before extension plateaus. Despite the external torque during swing, brief high-frequency bursts in the hamstrings and gastrocnemius reliably mark the stance-to-swing transition, while tibialis anterior activation and hamstring–quadriceps coactivation during mid-swing indicate residual voluntary effort (De Seta and Romeni, 2025).

The grand-averaged EMG envelopes shown in Figure 4 reveal clear, phase-dependent activation patterns across the six recorded lower-limb muscles and across both limbs. The observed temporal structure-proximal hip abductors/extensors active in early stance, quadriceps peaking at weight acceptance, and hamstrings/tibialis anterior increasing toward late stance and swing-matches canonical biomechanics of gait and supports the physiological validity of our recordings and preprocessing pipeline. The high repeatability of envelope shapes and peak timing across subjects and across left/right sides indicates that our gait-cycle segmentation and envelope extraction reliably capture functionally relevant motor commands rather than noise or artifact.

These EMG dynamics have several implications for interpretation of our decoding results and for controller design. First, the discrete, temporally localized bursts of EMG provide robust, high-signal-to-noise markers that are well suited for decisive, low-latency triggering: muscle envelopes rise sharply near the mechanical transition and therefore supply precise timing cues to confirm an intended transition that may be anticipated by EEG. This explains why EMG contributes to reducing false alarms and increasing the reliability of the hybrid detector even when EEG provides earlier but noisier anticipatory evidence. Second, the preserved relative timing between muscles across sides supports the use of multi-muscle feature sets (rather than single-muscle thresholds) and multimodal fusion: by combining temporal patterns across muscle groups the decoder can exploit redundancy to overcome local signal loss.

At the same time, the panel in Figure 4 also highlights potential sources of variability that likely impacted unimodal EMG performance (e.g., the low recall on the right limb). Although timing of peaks was largely consistent, there were modest amplitude differences between sides and occasional inter-subject variability in burst magnitude. Such amplitude differences can arise from electrode placement variability, inter-muscular crosstalk, anatomical differences, or subtle asymmetries in muscle recruitment strategy under assisted walking. Because our EMG normalization used per-subject, per-muscle calibration, amplitude fluctuations translate into changes in threshold crossing behavior and thus affect recall; this likely contributed to the lower EMG-only recall observed on the right limb in some participants.

EEG findings extend classical sensorimotor rhythm models to the assisted gait context (Pfurtscheller and Lopes Da Silva, 1999). During stance—when stability and weight-bearing are paramount-C3 exhibits a pronounced mu-band (8–13 Hz) event-related desynchronization(ERD) down to ~0.15 *μ*V^2^/Hz, potentially reflecting sensorimotor cortical involvement associated with gait-related motor preparation and postural regulation (Petersen et al., 2012; Wagner et al., 2016). Low-beta (14–20 Hz) power remains elevated (~0.18 *μ*V^2^/Hz), corresponding to tonic inhibition that prevents premature swing (Engel and Fries, 2010),while high-beta (21–30 Hz) shows modest suppression, consistent with its role in fine-tuning rather than gross support (Kilavik et al., 2009). Minimal theta (4–7 Hz) fluctuation (<0.05 *μ*V^2^/Hz) suggests little extra cognitive load once posture is maintained (Cavanagh and Frank, 2014). At swing onset-triggered either by muscle bursts or mu-ERD-the mu band rebounds toward baseline, indicating release of tonic drive. Simultaneously, both low- and high-beta bands exhibit strong event-related synchronization (ERS) up to ~0.18 *μ*V^2^/Hz, a hallmark of active movement execution and proprioceptive integration. The persistence of beta-ERS despite powered assistance may indicate continued cortical participation in movement regulation and proprioceptive integration during assisted locomotion (Engel and Fries, 2010).

By fusing mu-ERD’s anticipatory signal with beta-band confirmation and EMG onset timing, our BiLSTM decoder achieves high accuracy and responsiveness. The consistent interquartile ranges across legs and subjects demonstrate the reproducibility of these multimodal markers under exoskeleton load. Together, these phase-specific oscillatory and muscular signatures provide a physiologically grounded framework for robust real-time control, validating the selection of SMR and EMG features and highlighting the practical value of hybrid EEG–EMG integration for assisted locomotion (Artoni et al., 2017).

### Closed-loop control phase: comparison of EMG, EEG, and hybrid EEG–EMG conditions

Our findings consistently demonstrate that hybrid EEG–EMG control offers superior timing and efficiency over unimodal strategies. When driven solely by EMG, exoskeleton assistance exhibited the longest and most variable latencies (per-step mean ≈ 0.33 s left, 0.36 s right; range 0.10–0.75 s; Table 3), reflecting the stochastic nature of peripheral muscle activation and electrode crosstalk. Transitioning to EEG reduced latency variance (per-step mean ≈ 0.34 s left, 0.32 s right; range 0.28–0.40 s), indicating more consistent—but inherently slower—cortical ERD detection in *θ*/*μ*/*β* bands(Soekadar et al., 2015). By integrating EEG-derived sensorimotor features and EMG-derived neuromuscular features within a unified BiLSTM framework, the hybrid decoder achieved improved accuracy and responsiveness (per-step mean ≈ 0.29 s left, 0.28 s right; <0.15 s variance; Table 3). While measured per-step latency improvements were on the order of tens of milliseconds (hybrid vs. EMG mean difference ≈ 30–50 ms), the observed effect size (Cohen’s d ≈ 0.88) indicates a meaningful performance change at the system level. In a gait assistance context, earlier and more consistent triggering can reduce user waiting time at the 0° hold, smooth gait rhythm, and cumulatively shorten session durations (we observed ~2–3 min saved per 10-trial session and an overall ~7%–10% reduction in session time). Whether such millisecond-scale gains translate into clinically important outcomes (e.g., improved energy cost, gait symmetry, or effectiveness) remains to be established in patient cohorts; nonetheless, for real-time control, reductions of this magnitude meaningfully improve responsiveness and perceived system reactivity. Importantly, hybrid EEG–EMG fusion reduced triggering latency compared with both EEG-only and EMG-only approaches. Earlier detection of gait transitions may improve user-exoskeleton synchronization by allowing assistive actions to be initiated closer to the onset of the intended movement. Such improvements are particularly relevant for intention-driven exoskeleton control, where timely assistance is critical for achieving natural and responsive human-robot interaction. From an engineering perspective, reducing triggering latency may also enhance the transparency of human-robot interaction by minimizing the temporal mismatch between user intention and robotic assistance. This multimodal fusion not only minimized average delay but also narrowed inter-limb latency gaps to under 0.01 s and reduced inter-subject variability, thereby ensuring balanced bilateral assistance (Kilavik et al., 2009).

The paired subject-level analysis of task completion time (Figure 8) provides important functional context for the latency and decoding improvements observed under hybrid EEG–EMG control. While the absolute reduction in response latency between control modes was on the order of tens of milliseconds, the present results demonstrate that these seemingly modest temporal gains accumulated over repeated steps and translated into measurable reductions in total task duration during overground walking.

The consistency of the within-subject reductions across nearly all participants suggests that the observed benefit was not driven by isolated outliers but rather reflected a systematic improvement in timing reliability and intention-to-assistance coordination. Faster and more reliable detection of stance-to-swing transitions likely reduced hesitation periods at critical gait events, thereby smoothing the execution of each step and preventing small delays from propagating across successive strides. Over the course of ten 20-m outward-and-return trials, these incremental improvements accumulated into a clinically and functionally meaningful reduction in overall walking time.

Importantly, the paired representation also reveals reduced inter-individual variability under hybrid control compared to EMG-only or EEG-only modes. This finding suggests that multimodal fusion not only improves average performance but also enhances robustness across subjects with heterogeneous neuromuscular activation patterns. Such robustness is particularly relevant for future clinical translation, where signal quality and motor strategies may vary substantially across patient populations.

In summary, hybrid EEG–EMG control leverages complementary neural and muscular information to deliver fast, reliable, and efficient exoskeleton assistance. By reducing both per-step latency and overall trial duration, it advances the practical applicability of wearable robotic gait support in real-world—and ultimately clinical-settings.

The mean times for donning (13 min 17 s), setup (3 min 24 s), and doffing (3 min 0 s) the wearable exoskeleton—including the placement and removal of surface electrodes—compare favorably to previously reported ranges of 9–30 min for other lower-limb systems. Although our donning time appears longer than some reports, this reflects the added electrode preparation required for hybrid EEG–EMG control. Even so, the total procedure (mean ≈19 min) remains compatible with typical clinical session durations. These findings indicate that, with minimal extra burden for electrode application, the exoskeleton can be integrated into real-world workflows without undue time constraints, supporting its practical feasibility in routine therapy settings (Louie et al., 2021).

Our participants’ overall QUEST 2.0 satisfaction score—calculated as the sum of eight item ratings—averaged 32.0 ± 2.1 out of a maximum 40. This performance compares favorably with prior reports: EksoGT™ users scored 31.3 ± 5.7 and ReWalk users 29.4 ± 2.5, while Marsi Active Knee recipients averaged only 22.4 ± 3.2 (Cumplido-Trasmonte et al., 2024). On individual dimensions, our system’s Weight (Median 4.0; Mean 3.9 ± 0.7), Ease of Use (Median 4.0; Mean 3.8 ± 0.9), and Safety (Median 4.5; Mean 4.4 ± 0.8) all exceeded the Marsi Active Knee (Weight Median 2.8; Ease 2.6; Safety 3.6) and matched or outperformed the EksoGT™ device (Weight 3.8 ± 1.0; Ease 3.7 ± 0.9; Safety 4.3 ± 0.9). These high ratings confirm that users find the exoskeleton appropriately lightweight, straightforward to operate, and safe—a critical prerequisite for clinical adoption. Nonetheless, Comfort yielded the lowest mean score (3.7 ± 0.6), and some participants reported initial discomfort at the hip–seat interface. This was attributed to suboptimal hip-width adjustment during setup; after fine-tuning the seating fit, discomfort resolved and comfort scores improved. Additionally, the EEG cap required for hybrid control added pressure around the scalp, further impacting comfort ratings. These insights highlight opportunities to optimize the exoskeleton’s interface and electrode integration to enhance wearer comfort without compromising functionality. Overall, our QUEST 2.0 findings demonstrate strong user acceptance of the Exoskeleton’s core attributes while identifying practical refinements—particularly in EEG integration—to maximize comfort in future clinical implementations.

The present findings demonstrate that phase-dependent EEG and EMG features contain complementary information relevant to gait-event prediction. EEG-derived sensorimotor features contributed to intention-related gait transition detection within a multimodal real-time exoskeleton control framework. Hybrid decoding improved classification performance and reduced triggering latency compared with single-modality approaches, supporting the feasibility of multimodal intention-driven exoskeleton control. Future investigations should focus on validating the framework in diverse populations, assessing long-term robustness, and integrating adaptive control strategies toward more autonomous closed-loop exoskeleton assistance.

## Limitation

The present findings should be interpreted considering several methodological and practical constraints. The experimental protocol employed a fixed-order, within-subject design in which participants completed the EMG-only, EEG-only, and hybrid control conditions sequentially. Although standardized rest intervals were provided and trial order was included as a covariate in the analysis, residual learning or fatigue effects cannot be fully excluded. Future studies would benefit from a randomized or counterbalanced crossover design to more rigorously disentangle control-modality effects from order-related influences.

The study cohort was limited to a small group of seven healthy, right-handed adults. While sufficient for a proof-of-concept feasibility evaluation, this homogeneous sample restricts statistical power and limits generalizability. Responses to hybrid EEG–EMG control may differ substantially in clinical populations, such as individuals with stroke or spinal cord injury, who often exhibit altered neuromuscular coordination, asymmetric motor control, and increased signal variability.

User comfort also emerged as an important practical consideration. Several participants reported localized pressure at the hip–seat interface, primarily due to initial misadjustment of the exoskeleton’s hip-width settings, as well as scalp discomfort associated with prolonged EEG cap wear. These factors contributed to reduced comfort scores despite acceptable usability in other dimensions. Future work should therefore prioritize ergonomic refinements, including adjustable or cushioned seating interfaces, improved load distribution at the hip joints, and the adoption of dry or flexible EEG electrodes to reduce pressure points and enhance long-term wearability.

Although EEG-derived features contributed to the proposed multimodal framework, distinguishing genuine cortical activity related to movement intention from movement-related artifacts remains a critical limitation of the present study. ICA-based artifact attenuation was applied during offline EEG preprocessing to reduce ocular, muscular, and line-noise contamination for offline analyses; however, this procedure was not implemented in the online decoding pipeline because of real-time latency constraints. Instead, the online system relied on causal filtering and real-time feature extraction. Hip kinematics were used only for gait-event labeling and were not included as BiLSTM input features, preventing direct utilization of mechanical trajectories by the classifier. In addition, the hybrid framework incorporated EMG information, providing complementary neuromuscular information. Nevertheless, movement-related artifacts and neural activity may partially overlap temporally during locomotion, and scalp EEG alone cannot separate these contributions. Future studies incorporating online artifact correction, source localization, artifact-resistant mobile EEG systems, and additional physiological validation approaches are required to further investigate the neural specificity of EEG-derived features. However, real-time artifact rejection methods, such as online ICA or adaptive artifact cancellation, were not implemented in the present system because they may introduce additional computational latency that could compromise real-time triggering performance. Similarly, source localization analysis was not performed due to the limited number of scalp EEG channels and the focus of the present study on system-level intention-related decoding. Future studies should incorporate high-density mobile EEG, online artifact correction algorithms, source-level analysis, and additional physiological measurements to further validate the cortical origin of EEG-derived features during locomotion. From a real-time decoding perspective, the use of a fixed-length temporal window introduces an inherent observation delay, which was incorporated into the reported triggering latency. Although this window-based strategy enables temporal feature integration for BiLSTM decoding, future studies may investigate causal recurrent architectures, adaptive streaming models, or more efficient online decoding strategies to further reduce computational and observation delays. Furthermore, evaluation in larger clinical cohorts and integration with adaptive control strategies will be essential for establishing long-term closed-loop exoskeleton assistance.

## Conclusion

This study demonstrates the feasibility of multimodal EEG–EMG fusion for real-time gait-intention detection and exoskeleton triggering. The proposed BiLSTM framework achieved improved decoding accuracy and reduced response latency relative to unimodal approaches, suggesting that multimodal neural-muscular integration may provide an effective strategy for intention-aware exoskeleton control.

## Data Availability

The raw data supporting the conclusions of this article will be made available by the authors, without undue reservation.
